# Supplementary material for: Global impacts of future cropland expansion and intensification on agricultural markets and biodiversity
Source: Nat Commun. 2019 Jun 28;10:2844. doi: 10.1038/s41467-019-10775-z (PMC6598988; doi:10.1038/s41467-019-10775-z)
Supplement: Supplementary file 1 — Supplementary Information [file 41467_2019_10775_MOESM1_ESM.pdf]

Supplementary Information for

Global impacts of future cropland expansion and intensification
on agricultural markets and biodiversity

Zabel et al.

This PDF file includes:

Supplementary Notes 1 to 8

Supplementary Figures 1 to 23

Supplementary Tables 1 to 5

Supplementary References

Table of Contents

Supplementary Note 1: Models 2
PROMET 2
DART-BIO 2
Description of Scenarios 3
Supplementary Note 2: Calculation of integrated expansion potential 5
Biophysical expansion potential 5
Consideration of socio-economic conditions 7
Integrated expansion potential 7
Integration of cropland expansion into DART-BIO 8
Supplementary Note 3: Calculation of integrated intensification potential 11
Biophysical intensification potentials 11
Integrating intensification potentials 12
Supplementary Note 4: Impacts on agricultural markets under the expansion and intensification scenarios 16
Supplementary Note 5: Hotspot Analysis 21
LISA Analysis 22
Quantile Overlay Analysis 23
Supplementary Note 6: Overlap of expansion and intensification potentials with protected areas 28
Supplementary Note 7: Policy Scenario: Impact of excluding protected areas from expansion potentials on agricultural markets 29
Supplementary Note 8: Data sources 32

Supplementary Note 1: Models

PROMET

PROMET is a hydrological land surface process model, which has been extended by a biophysical dynamic vegetation component to model crop growth and yield formation[1-4]. It uses first order physical and physiological principles to determine net primary production and respiration based on approaches from Farquhar et al.[5] and Ball et al.[6], combined with a phenology and a two-layer canopy architecture component of Yin and van Laar [7]. PROMET takes into account the dependency of net primary production and phenology on environmental conditions including meteorology, CO_2 concentration for C3 and C4 pathways as well as water and temperature stress. The mass and energy balance of the canopy and underlying soil surface are iteratively closed for each hourly simulation time step. The canopy and phenology component allocates assimilates into the different plant organs of the canopy depending on the phenological stage of development. Assimilates that are accumulated within the fruit fraction during the growing period determine the dry biomass available for yield formation. The simulation is performed on an hourly time step to account for non-linear reactions of crop growth to environmental conditions (mainly light, water, temperature and wind). Depending on the reaction of the considered crop to meteorological and soil-specific conditions, the crop may either die due to water, heat or cold stress before being harvested or it may not reach maturity. In both cases, this results in total yield loss.

DART-BIO

The Dynamic Applied Regional Trade (DART) model is a multi-sectoral, multi-regional recursive-dynamic computable general equilibrium (CGE) model of the world economy[8,9]. The DART model is based on the Global Trade Analysis Project (GTAP) database covering multiple sectors and regions. The economy in each region is modelled as a competitive economy with flexible prices and market clearing conditions. Economies are connected via bilateral trade flows. Hence, market feedbacks of commodities and regions are taken into account. According to microeconomic theory, producers maximize profits, consumers their utility. Consumers are modelled via representative households. They receive income generated by providing primary factors to the production process. Disposable income is used for maximizing utility by purchasing goods. DART-BIO has non-unitary income elasticities by using the linear expenditure system (LES) approach[10].

The version DART-BIO is calibrated based on the GTAP8.1 database[11], which represents the global economy in 2007 and covers 57 sectors and 134 regions. The DART-BIO model considers 23 regions, 38 sectors, 45 products (Supplementary Table 2) and 21 factors of

production. Land is one of the production factors. We use the GTAP-AEZ database which divides land into 18 so-called agro-ecological zones (AEZs)[12]. A detailed description of the models is available in[13,14]. Within each AEZ and region, land is allocated to different uses (i.e. cropland, pasture and forest) via a constant elasticity of transformation (CET) structure. In annual time steps, DART-BIO simulates runs until 2030. The dynamics of the model are driven by exogenous driving forces: The saving rate and the gross rate of return on capital determine capital accumulation. The capital stock of the next period is altered by the current period's investments and depreciation, while the allocation of capital among sectors is caused by the intra-period optimization of the firms. Regional households are characterized by a constant savings rate over time. Labor supply and productivity are determined by changes in labor force, the rate of labor productivity growth, and the change in human capital accumulation. Labor productivity improvement rates and growth rates of human capital are constant, but regionally different. The model assumes growth rates of the labor force according to projections of participation rates taken from the PHOENIX model[15] and in line with OECD projections[16]. Population growth is taken from the United Nations, Department of Economic and Social Affairs, Population Division[17].

CGE models are rarely validated. Van Dijk et al.[18] state that "Curiously, however, in contrast to modelling efforts in, for example, the biophysical sciences, CGE model findings are seldom subjected to any systematic validation procedure. A cursory review of the literature reveals isolated single country CGE model validation exercises, although with a dearth of available data, there is a paucity of equivalent studies which implement such a procedure in a global CGE context". While there is not systematic validation procedure performed for the DART-BIO model, the results of the reference run are compared to other business as usual studies such as the OECD/FAO agricultural outlook. Further, the modelling team is engaged in an initiative by the Global Trade Analysis Project that aims to develop best practices for baseline generation.

In this study, DART-BIO is used to calculate the impact of cropland expansion and intensification on agricultural markets taking repercussions of international markets across sectors and regions into account.

To capture the population dynamics and income growth, we compare the situation in 2030 under a cropland and an intensification scenario to a reference scenario.

Description of Scenarios

The **reference scenario** carries on current trends and developments. Socio-economic trends and developments are affected by the following assumptions:

a) Consumer demand: Household's disposable income is used for maximizing utility by purchasing goods. With the LES approach[10] a representative consumer is split into two categories: a 'subsistence consumer' and a 'surplus consumer'. This implies that a household spends a fixed part of its income for a subsistence quantity, while the rest is spent according to elasticities of demand. The higher income elasticities of demand for a certain good are, the stronger is the demand for that good with rising incomes relative to demand for other goods. The income elasticities are taken from the GTAP database.

b) Land endowment: We use a CET function that restricts the mobility of land from one of these land types to another economic use[19-21]. We choose a three-level nesting where land is first allocated between land for agriculture and managed forest. Then, agricultural land is allocated between pasture and crops. In the third level, cropland is allocated between rice, palm, sugar cane/beet and annual crops (wheat, maize, rapeseed, soybeans, rest of cereal grains, rest of oilseeds and rest of crops). At each level, the elasticity of transformation increases, replicating that land is more mobile between crops than between forestry and agriculture. The total land endowment (sum of all managed land) is fixed under the reference scenario. Hence, we assume no cropland expansion into unmanaged areas.

c) the development of land productivity: The change in output per unit of land input is determined by the total factor productivity, the characteristics of agricultural production functions, and changing yields under climate change. In the agricultural production functions of DART-BIO, we use lower elasticity of substitution between land and other factors of production (0.2-0.25) compared to other global models (e.g. 0.8 in the AIM model, 0.5 in the ENVISAGE model[22]). Yield changes are taken from Delzeit et al.[23].

d) Policies: In all scenarios, we adopt taxes and subsidies that producers and consumers face based on the GTAP database. The database includes domestic taxes, primary factor and commodity tax rates, firms domestic and import taxes, governmental domestic and import tax rates, import taxes for final consumption, and import taxes for all commodities and regions. In addition, we take biofuel policies into account. Shares of biofuels on transport fuels are taken from OECD/FAO[24], and for EU member states from Beurskens et al.[25].

The **expansion scenario** adopts all reference assumptions except those on land endowment. We assume that the top 10% (7,340,304 km²) of the integrated potential expansion area (see Supplementary Note 2) is available for agricultural production in addition to the land endowment in DART-BIO as in the reference scenario. The calculation of this potential and the implementation into DART-BIO are explained in detail in Supplementary Note 2.

The **intensification scenario** adopts all reference assumptions except the development of land productivity. We introduce a partial closing of yield gaps by reducing regional yield gap ratios

(potential agro-economic yields divided by the statistical yield) in order to meet the same production quantity as under the expansion scenario. A detailed description is available in Supplementary Note 3.

Supplementary Note 2: Calculation of integrated expansion potential

We calculated a potential for cropland expansion, based on future climate conditions. Subsequently, we integrated socio-economic drivers of future land use change. The integrated cropland expansion was then used for two analyses: the simulation of the impact of cropland expansion on agricultural markets, and the spatial association of expansion potentials and endemism richness.

Biophysical expansion potential

We applied an updated version of the biophysical potential for expansion, as described in Delzeit et al.[26]. The biophysical potential for expansion (Supplementary Fig. 1) is an index between 0 and 100, determined by the crop suitability approach based on Zabel et al.[27] and the available land for conversion into cropland[26]. Available land for conversion in this context includes all suitable land that is not yet under cultivation[28] or urbanized (according to ESA-CCI land use/cover classification)[29], since a conversion of urban areas into cropland can be considered as unrealistic.

Supplementary Table 1: Considered crops for simulation of biophysical expansion potential and biophysical intensification potential.

Cassava (*Manihot esculenta*)
Groundnut (*Arachis hypogaea*)
Maize (*Zea mays*)
Millet (*Pennisetum americanum*)
Oil palm (*Elaeis guineensis*)
Paddy rice (*Oryza sativa*)
Potato (*Solanum tuberosum*)
Rapeseed (*Brassica napus*)
Rye (*Secale cereale*)
Sorghum (*Sorghum bicolor*)
Soy (*Glycine maximum*)
Sugar beet (*Beta vulgaris subsp. vulgaris*)
Sugarcane (*Saccharum officinarum*)
Barley (*Hordeum vulgare*)
Sunflower (*Helianthus annus*)
Summer wheat (*Triticum aestivum*)
Winter wheat (*Triticum aestivum*)

The crop suitability represents the suitability of 17 crops (Supplementary Table 1) to grow under the prevailing local natural conditions. We considered climate conditions (temperature, precipitation, solar radiation), soil properties (texture, proportion of coarse fragments and gypsum, base saturation, pH content, organic carbon content, salinity, sodicity), and terrain (elevation, slope) for determining crop suitability. The crop suitability was calculated globally at 30 arc seconds spatial resolution for each crop. Climate data for 2011-2040 was used from high-resolution T213 runs of the general circulation model ECHAM5 of the Max-Planck Institute for Meteorology (MPI-M)[30-32]. The runs were driven with SRES A1B emission scenario conditions. We applied a bias correction on the climate data, based on monthly derived factors from the WordClim dataset[33] that were interpolated to daily factors. Soil parameters were gathered from the harmonized world soil database v1.21 (HWSD)[34]. Topography data was derived from Space Shuttle Topography Mission (SRTM) data[35]. Currently irrigated areas according to Meier et al.[36] were considered in the crop suitability approach by assuming no water limitation on these areas. We assumed that irrigated areas do not change until 2030. The resulting crop suitability for each crop was aggregated to an overall suitability, taking at each pixel the value of the most suitable crop. When compiling the expansion potential, the overall suitability was aggregated from 30 arc seconds to 5 arc minutes to be at the same consistent scale as the available land-use information[28].

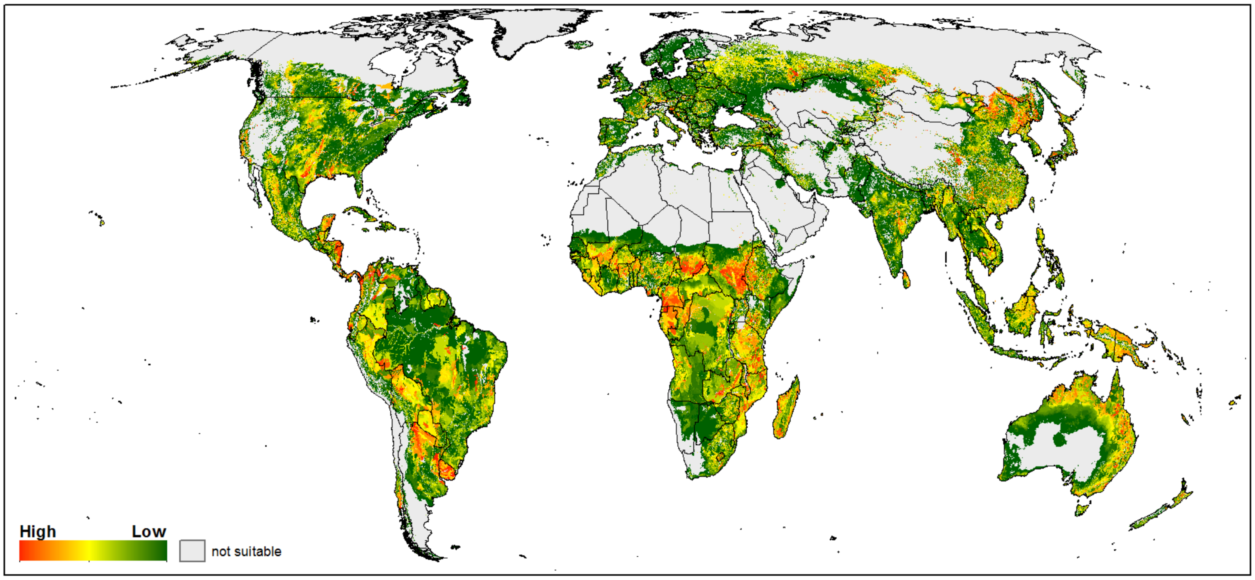

Supplementary Figure 1: Biophysical potential for cropland expansion. It is calculated from available land for conversion and biophysical suitability for 17 crops under near future climate change conditions (2011-2040). Grey areas indicate no suitability for expansion. High values indicate highly suitable land that is not yet used as cropland.

Consideration of socio-economic conditions

The expansion of cropland is largely driven by future investment decisions and not only restricted by biophysical conditions, but also by the availability of infrastructure and legal or cultural factors. Since there is no common agreement on these drivers and a lack of globally available data on these socio-economic factors, we used data on annual growth rates of arable land according to Alexandratos and Bruinsma[37] to integrate socio-economic conditions into the expansion potentials. The data is available for administrative regions. Taking share of land endowment by agro-ecological zones on the total land endowment of a region from the DART-BIO model, we spatially disaggregated this data into 414 sub-regions (Supplementary Fig. 2).

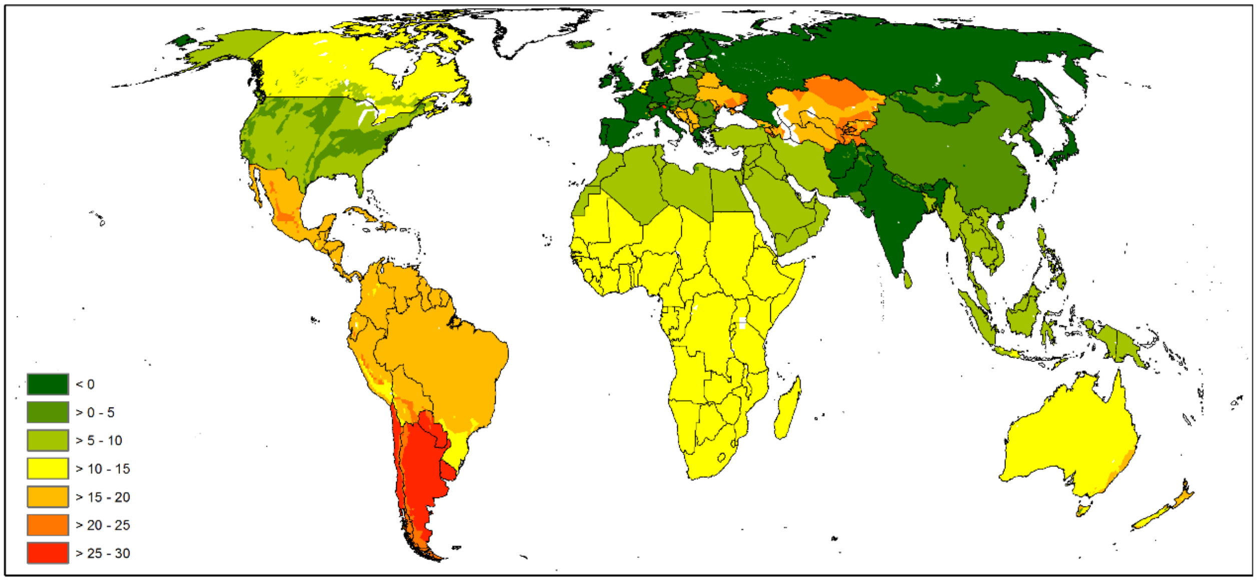

Supplementary Figure 2: Percentage change in cropland by 2030 compared to 2007 (socio-economic expansion). The data is based on Alexandratos and Bruinsma [37] and spatially refined with DART-BIO.

Integrated expansion potential

We weighted the socio-economic expected expansion with the biophysical potentials for expansion to obtain the integrated expansion potential. As a result, high values were assigned to regions that are biophysically highly suitable for expansion and accordingly show high expected socio-economic expansion rates until 2030, while regions with low values are either only marginally suitable for expansion or not expected to expand due to socio-economic aspects (Supplementary Fig. 3). The integrated potential for cropland expansion was set to zero in regions where cropland is expected to decrease.

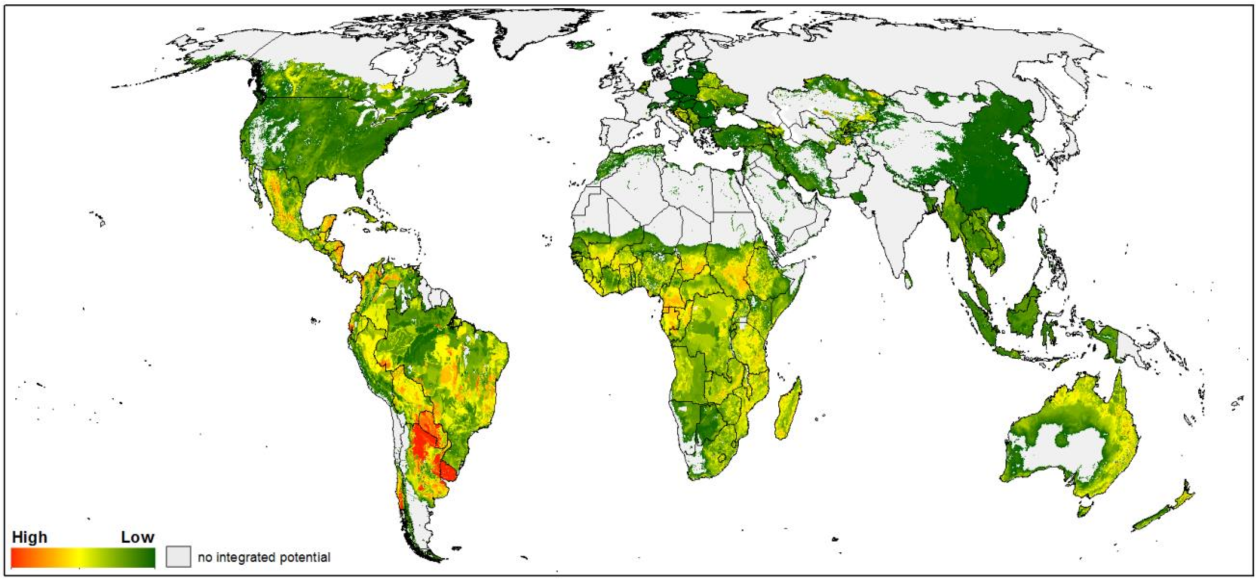

Supplementary Figure 3: Integrated potential for cropland expansion by 2030. Grey areas have no integrated potential for expansion either due to biophysical reasons or since expansion is not expected to happen because of socio-economic reasons**.**

Integration of cropland expansion into DART-BIO

To determine the impact of cropland expansion on agricultural markets with DART-BIO, we defined a cropland expansion scenario and compared the results to the reference scenario (see Supplementary Note 1). In this scenario, we assumed that the top 10% (7,340,304 km²) of the integrated potential expansion area was available for agricultural production in addition to the land endowment in DART-BIO as in the reference scenario. Land endowment in DART-BIO is in value units, the expansion area in km^2. Supplementary Fig. 4 shows the regions where the area of the top 10% integrated expansion potential is distributed. Most of the area was found in Sub-Saharan Africa (2,532,755 km²), followed by Rest of Latin America (1,573,803 km²), the countries of Paraguay, Argentina, Uruguay and Chile (1,337,796 km²), and Brazil (1,124,618 km²). Middle and South America together with Sub-Saharan Africa make almost 90% of the top 10% area with the highest integrated expansion potential.

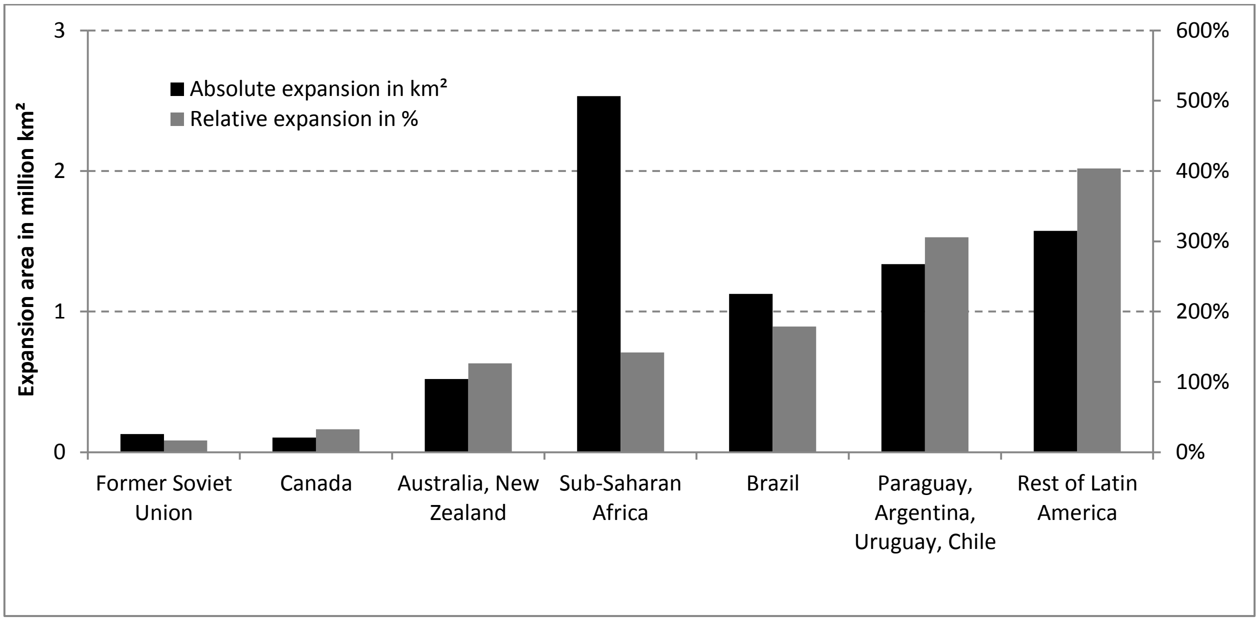

Supplementary Figure 4: Summed cropland expansion area by world region. Black bars represent the absolute cropland expansion in top10% areas in million km^2. The relative expansion (grey bars) displays the change in expansion in 2030 relative to the starting value of cropland in 2007. While the highest absolute expansion occurs in Sub-Saharan Africa, the highest relative expansion takes place in South- and Central America. The relative expansion (compared to 2007) is important to understand the changes between the reference scenario and the expansion scenario.

The land endowment (value of land, vfm) consists of a price of land times the area for each crop, region, and AEZ (Supplementary Equation (1)). The land endowment in 2030 including the expansion area (E) by region and AEZ is defined in Supplementary Equation (2). Supplementary Equation (3) depicts the value of land over time in $ that was implemented into the DART-BIO model.

$$\mathrm{vfm}_{\mathrm{c,r,l}} = P_{c,r,l} * A_{c,r,l}$$ Supplementary Equation (1)

$$\mathrm{vfm2030}_{\mathrm{r,l}} = \sum_c P_{c,r,l} * \left(\sum_c A_{c,r,l} + E_{r,l}\right)$$ Supplementary Equation (2)

$$\mathrm{vfmY}_{\mathrm{y,c,r,l}} = \mathrm{vfmY}_{\mathrm{y-1,c,r,l}} + \left(\mathrm{vfmY}_{\mathrm{y,c,r,l}} * \left(\sum_c \frac{\mathrm{vfm2030}_{\mathrm{r,l}}}{\mathrm{vfm}_{\mathrm{c,r,l}}}^{\frac{1}{\mathrm{yr}}} - 1\right)\right)$$ Supplementary Equation (3)

With:

$\mathrm{vfm}_{c,r,l}$	value of land ($)
yr	total number of years (2030-2007=23)
$A_{c,r,l}$	area (km^2)
y	years (2007-2030)
$P_{c,r,l}$	price of land ($/$km^2$)
c	crop

$E_{r,l}$ Expansion area, top 10 expansion (km_2)

r regions

$\mathrm{vfm2030}_{\mathrm{r,l}}$ value of land in 2030 ($)

l index of agro-ecological zone

$\mathrm{vfmY}_{\mathrm{y,r,l}}$ value of land over time ($)

One model output is the land use by activity (crops, pasture, forest) in values ($). Changes in land used by crops of model simulations were determined by changes of the exogenous expansion (vfmY) and endogenous changes in the land use in the CET function. To calculate the changes in harvested areas, we applied Supplementary Equation (4):

$$\Delta \mathrm{HE}_{\mathrm{y,r,l}} = \mathrm{HE}_{\mathrm{y,r,l,c}} + \mathrm{HE}_{\mathrm{y,r,l,c}} \left(\frac{\sum_c A_{c,r,l}}{\sum_c A_{c,r,l} + E_{r,l}}^{\frac{1}{yr}} - 1 \right) \quad \text{Supplementary Equation (4)}$$

$\Delta \mathrm{HE}_{\mathrm{y,r,l,c}}$ Change in harvested area with top 10 expansion (%)

$\mathrm{HE}_{\mathrm{y,r,l,c}}$ Harvested area (km^2)

$\mathrm{HE10}_{\mathrm{y,r,l,c}}$ Harvested area with top 10 expansion (km^2)

$\mathrm{LE}_{\mathrm{y,r,l,c}}$ Shares of land endowment

$\Delta \mathrm{LE}_{\mathrm{y,r,l,c}}$ Change shares of land endowment (%)

Supplementary Note 3: Calculation of integrated intensification potential

Biophysical intensification potentials

The crop growth model PROMET globally simulates potential yields (see Supplementary Note 1). We used the same ECHAM5 climate model data for driving the PROMET model as described in Supplementary Note 2. The 6-hourly climate dataset (temperature, precipitation, direct and diffuse short wave radiation, long wave radiation, surface pressure, relative humidity and wind speed) was temporally interpolated to an hourly time step.

For identifying intensification potentials (Supplementary Fig. 5) on existing cropland, we simulated potential yields, assuming a perfect management of crops. This implies that no nutrient stress, pests and diseases occur during crop growth and that optimal sowing dates and potential number of harvests per year, adapted to the climate conditions for 2011-2040, are applied from globally derived data on the optimal start of the growing season from Zabel et al.[27], for each of the 17 considered crops (Supplementary Table 1). We assumed that cultivars are adapted to the changed temperature conditions in 2011-2040 by adjusting phenological speed for each location to the same length between harvest and maturity as in the reference simulation from 1981-2010. The adjustment takes place for climate averages and not annually, since we assume that farmers select cultivars on basis of a long-term practical experience, and thus are not immediately adapting new cultivars. For the PROMET potential yield simulation, we applied a sampling approach described in Mauser et al.[2]. We used a statistically representative set of approx. 250,000 samples that were randomly distributed on the Earth's agriculturally suitable land surface. The simulation was carried out for all crops at each of the samples for rainfed and irrigated conditions separately. We used data on current harvested[28] and irrigated areas[38] to determine areas that are available for intensification and to weight irrigated and rainfed yields according to the proportion of current irrigated and rainfed areas. Crop production was then aggregated for 414 sub-regions. The model has been validated at field scale[1], and simulated global yields have been compared against statistical values and statistical models on a country level[2].

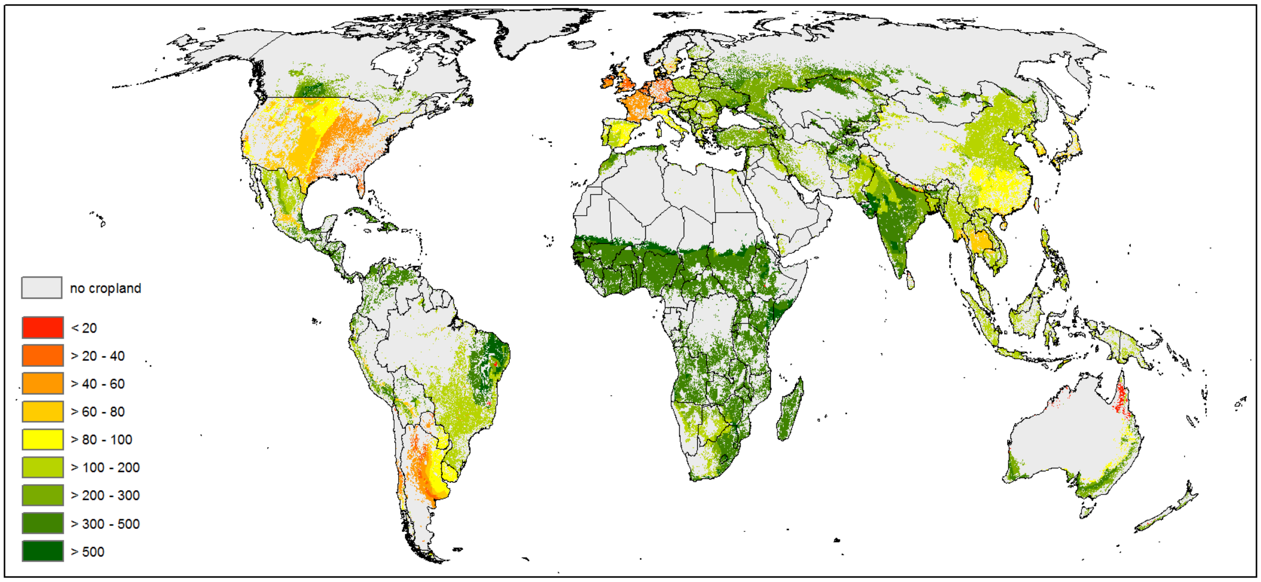

Supplementary Figure 5: Biophysical potential for intensification (2011-2040) in percentage change compared with today's production. The different crops are area-weighted averaged. Map shows potential for intensification on todays cultivated area where the fraction of cropland per pixel is greater than 0%.

Integrating intensification potentials

Following Mauser et al.[2] the potential yields of 17 crops as an output of PROMET were spatially aggregated to the 23 regions applied by DART-BIO and grouped into 10 crop categories (Supplementary Table 2) to match with the crop categories used in DART-BIO.

The marginal profitability of different crops at different locations was used to allocate crops by maximizing profit within a region. The marginal profitability depends on socio-economic scenarios that were implemented into the DART-BIO model (Supplementary Fig. 6). The allocation resulted in integrated potential yields that fed back to the DART-BIO model in terms of changed agricultural productivities, which in turn altered the relative profitability of crops, such that the re-allocation was repeated iteratively until a stable allocation was established. The coupling approach is based on Mauser et al.[2] and allows for taking into account that land allocation to crops changes over time due to e.g. changing cropping decision of farmers, food consumption behavior, climate change or technological progress.

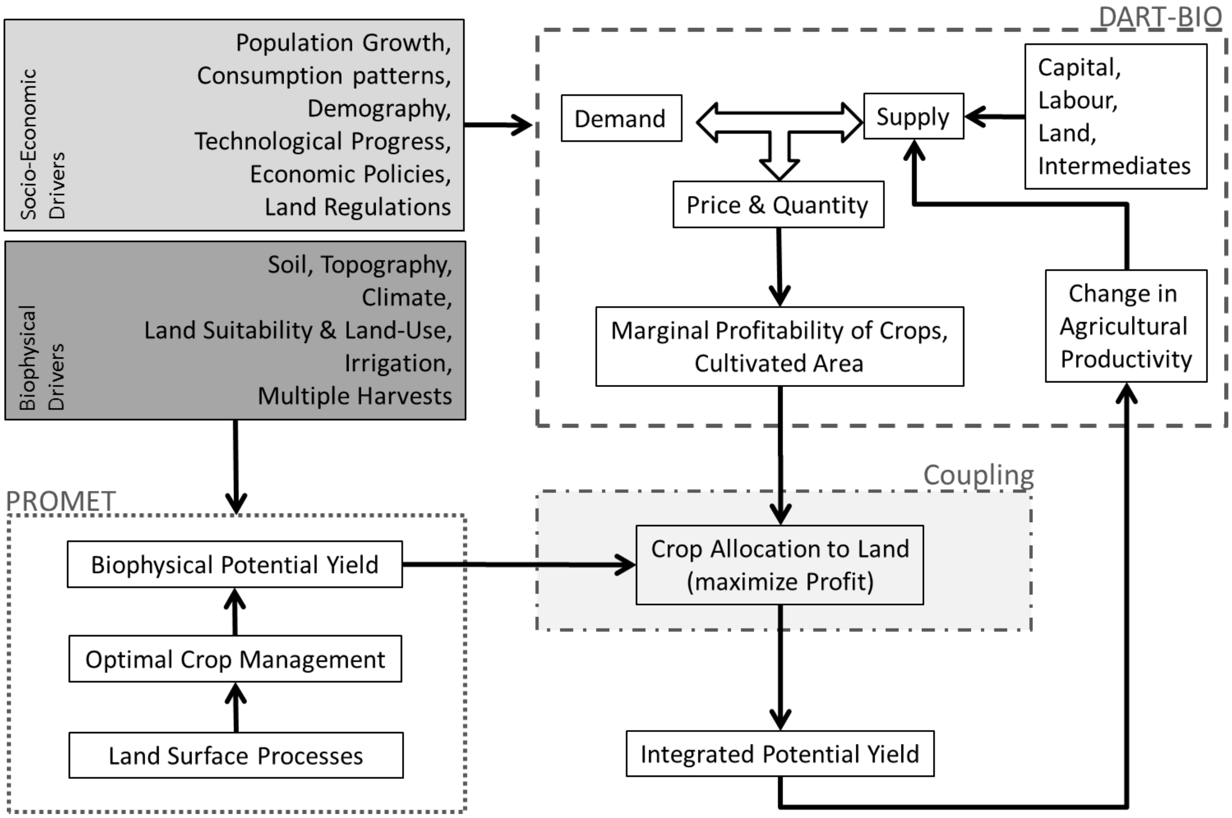

Supplementary Figure 6: Coupling approach between PROMET and DART-BIO, based on Mauser et al.[2].

The outcome was an integrated potential for intensification (Supplementary Fig. 7). It represents the potential yield on today's cultivated cropland areas[28] if perfect crop management was utilized under market-oriented conditions. Compared to statistical yields[39], the yield gap describes the difference between the potential yield and the statistical yield for each crop and region. The yield gaps were closed in equivalence to the expansion scenario on the 10% areas that show the highest yield gap. Since the closure of the yield gap is made more difficult with its decline because exponentially more input is required, it can be assumed that the top 10% areas showing the highest yield gaps are the economically most efficient areas globally available for intensification[23].

In order to produce comparable scenarios, and since a complete closure of the yield gap is unrealistic, we closed the yield gap by 28% for each region so that the same global production was reached as in the expansion scenario.

Supplementary Table 2: List of sectors (industries and products (goods)) in DART-BIO.

**Agricultural related products (29)**		
**Crop Category**		**Representation in PROMET**
PDR	Paddy rice	Rice
WHT	Wheat	Summer wheat, winter wheat
MZE	Maize	Maize
GRON	Rest of cereal grains	Sorghum, millet, rye, barley
C_B	Sugarcane, sugar beet	Sugarcane, sugar beet
SOY	Soybean	Soy
PLM	Oil palm fruit	Oil palm
RSD	Rapeseed	Rapeseed
OSDN	Rest of oil seeds	Groundnut, sunflower
AGR	Rest of crops	Cassava, potato, maize silage
**Processed**		
DDGSg*	DDGS from other cereal grains	
DDGSm*	DDGS from maize	
DDGSw*	DDGS from wheat	
FOD	Rest of food	
FRI	Forest related industry	
FRS	Forestry	
ILVS	Indoor livestock	
OLVS	Outdoor livestock	
OSDNmeal*	Meal from other oil seeds	
OSDNoil*	Oil from other oil seeds	
PCM	Processed animal products	
PLMmeal*	Palm meal	
PLMoil*	Palm oil	
RSDmeal*	Rapeseed meal	
RSDoil*	Rapeseed oil	
SGR	Sugar	
SOYmeal*	Soybean meal	
SOYoil*	Soybean oil	
VOLN	Other vegetable oils	
**Energy products (13)**		
BDIE	Biodiesel	
BETH	Bioethanol	
COL	Coal	
CRU	Oil	
ELY	Electricity	
ETHG*	Bioethanol from other grains	
ETHM*	Bioethanol from maize	
ETHS	Bioethanol from sugar cane	
ETHW*	Bioethanol from wheat	
GAS	Gas	
MDIE	Motor diesel	
MGAS	Motor gasoline	

OIL	Petroleum and coal products
**Non-energy products (3)**	
CRPN	Other chemical rubber plastic prods
ETS	Paper, minerals and metals
OTH	Other goods and services

*: All goods are produced by an analogous industry, except were indicated by an asterisk (*), which indicates jointly produced goods. Bioethanol and Dried Distiller Grains with Soluble (DDGS) are jointly produced by the bioethanol industry (3 types of industries); and oilseeds oil and meal are jointly produced by the vegetable oil industry (4 types of industries).

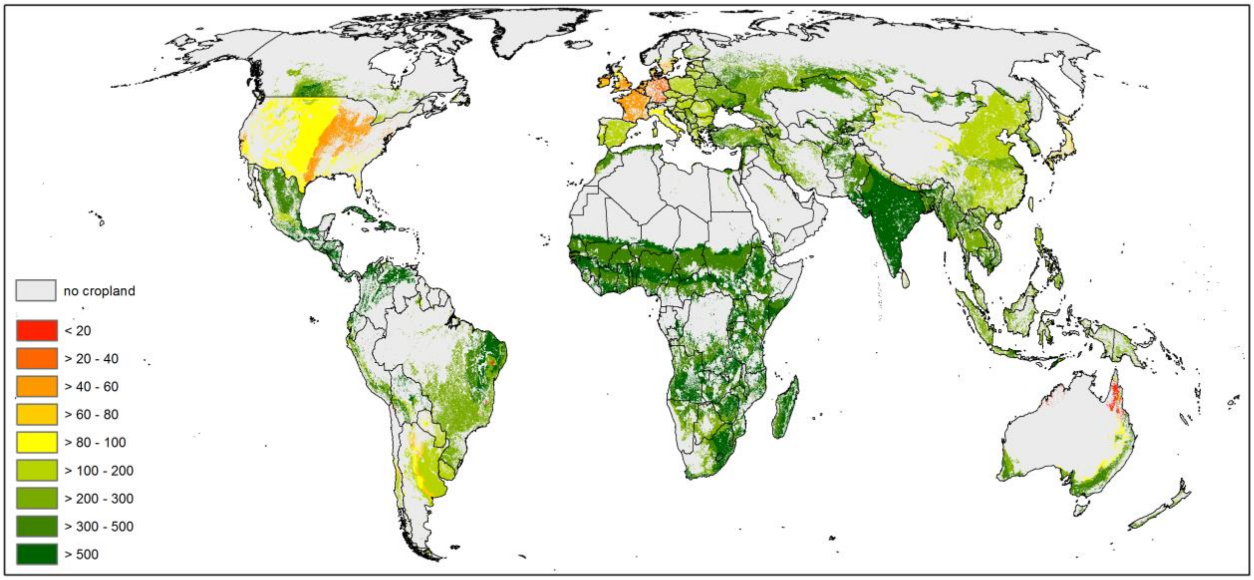

Supplementary Figure 7: Integrated potential for intensification in percent of potential yield increase compared to statistical yields. The 17 different crops are averaged for illustration purposes by weighting the yields by crop-specific area.

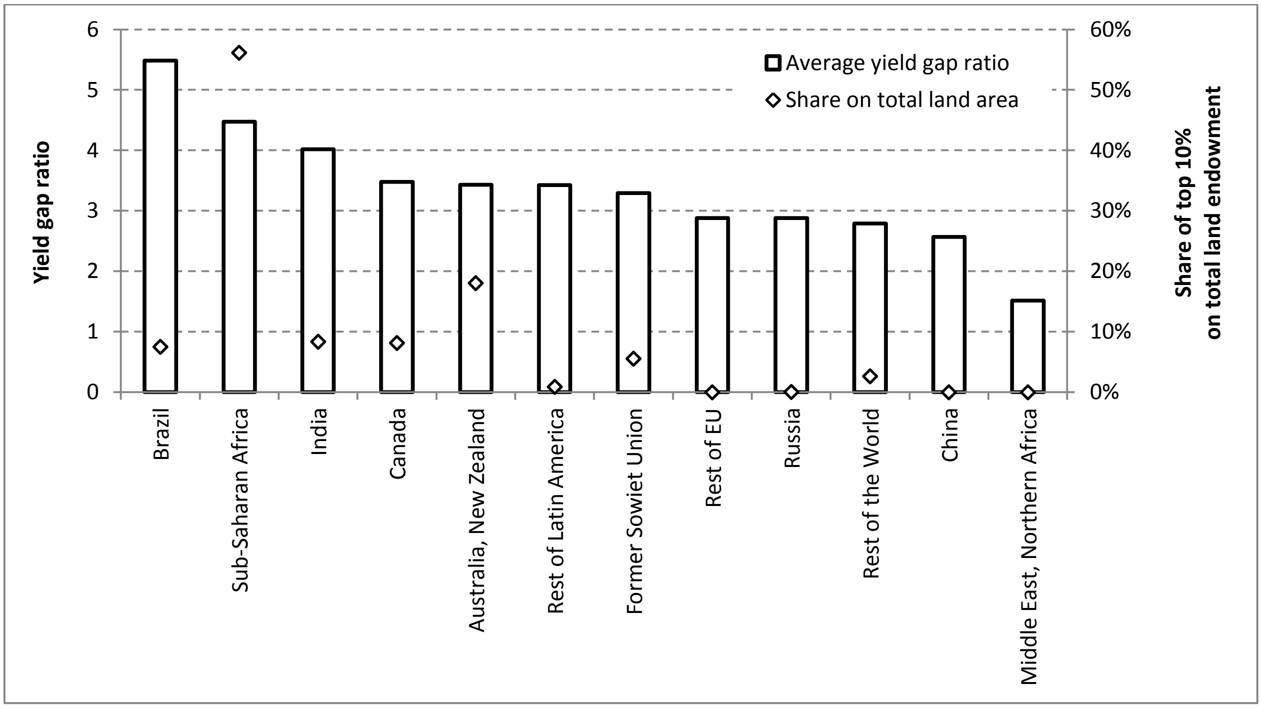

Supplementary Figure 8: Integrated yield gap ratios (potential yield / statistical yield) and share of areas with top 10% intensification potential on total land endowment.

Supplementary Note 4: Impacts on agricultural markets under the expansion and intensification scenarios

Under the land expansion scenario, the production of sugar cane, rest of oil seeds, and soybeans increased strongest amongst the crops (Supplementary Fig. 9). They were produced in regions with high integrated expansion potentials. Sugar cane production was biggest in South and Central America, India and Sub-Saharan Africa (Supplementary Fig. 10). The land expansion scenario caused a more than doubling of South- and Central American sugar cane production. We saw also a strong increase in production of soybeans in Central- and South America. With soybeans being a crop with high share of trade on production, the changes had impacts on other countries: production in the USA declined (Supplementary Fig. 11). The area not used for soybean production in the USA was used for different grains (Supplementary Figs. 12, 13, 14). An interesting case was Sub-Saharan Africa. One would expect that due to its high integrated expansion potentials, the increase in crop production of e.g. wheat by 184% would significantly affect the global wheat market. But with a share of only 5% in total global production, other regions were hardly affected by changes in Sub-Saharan production (Supplementary Fig. 12).

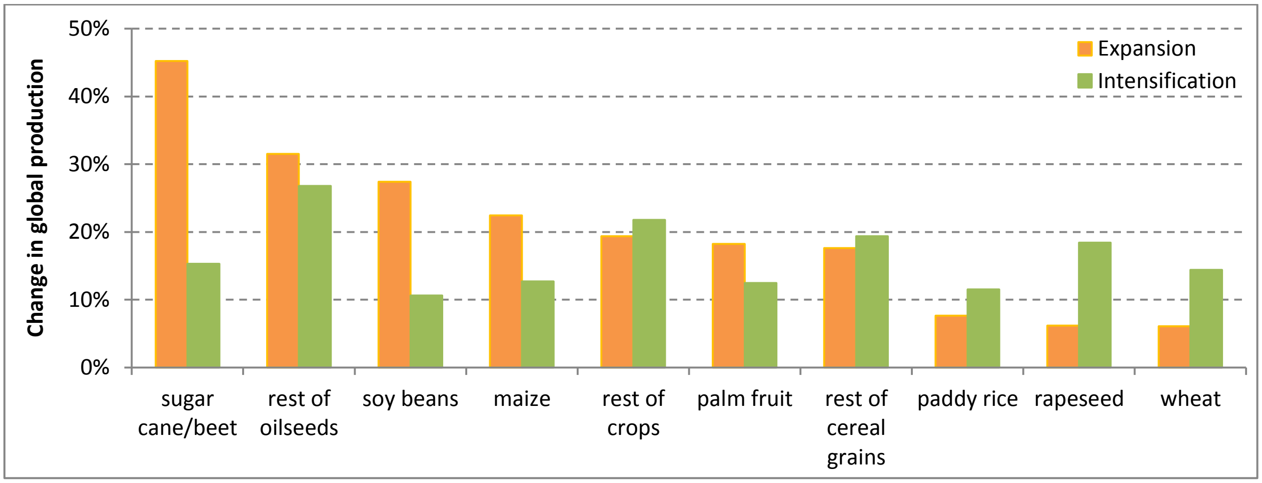

Supplementary Figure 9: Percentage change in global production of 10 crops under the expansion and intensification scenario in 2030 compared to the reference scenario in 2030.

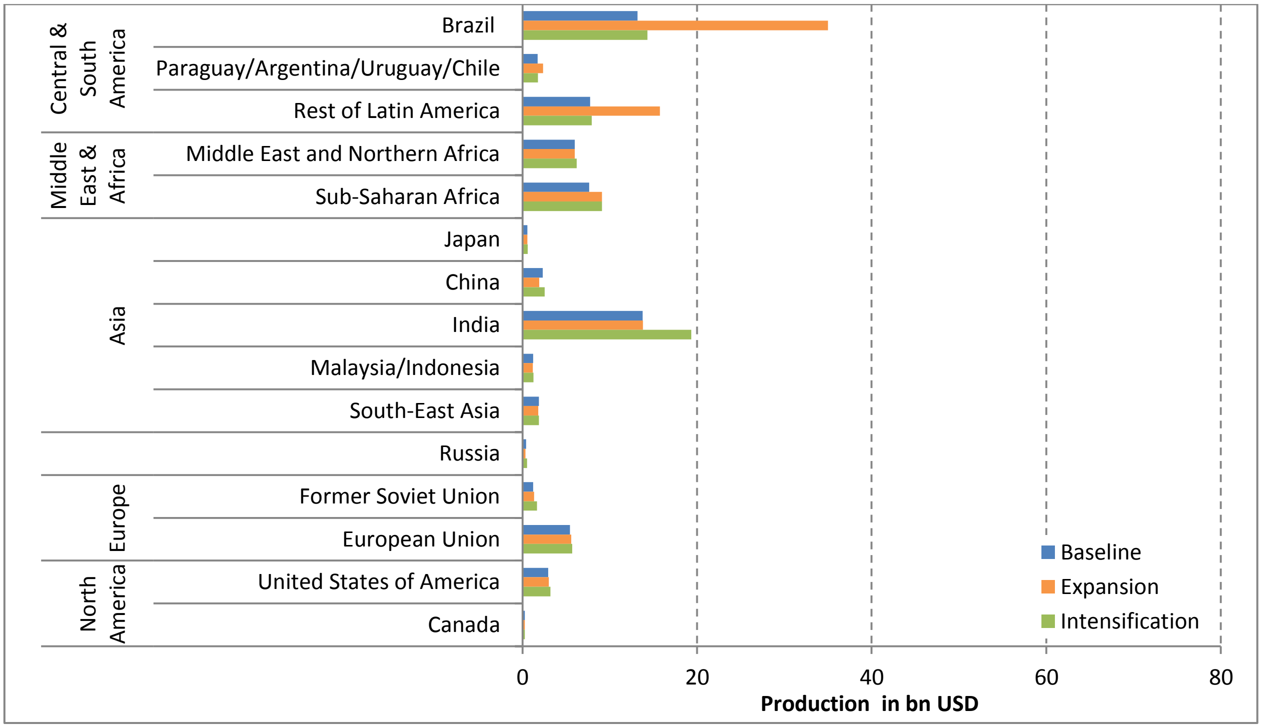

Supplementary Figure 10: Production of sugar cane / beet in 2030 for the three scenarios.

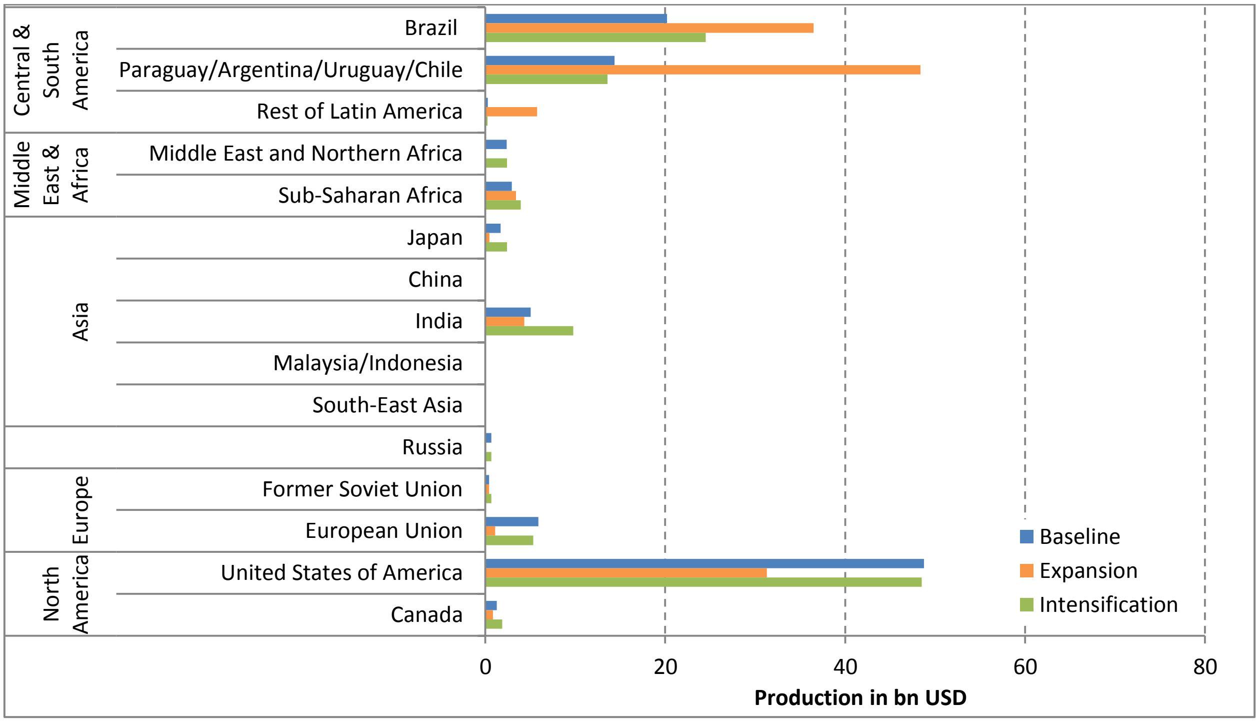

Supplementary Figure 11: Production of soybeans in 2030 for the three scenarios.

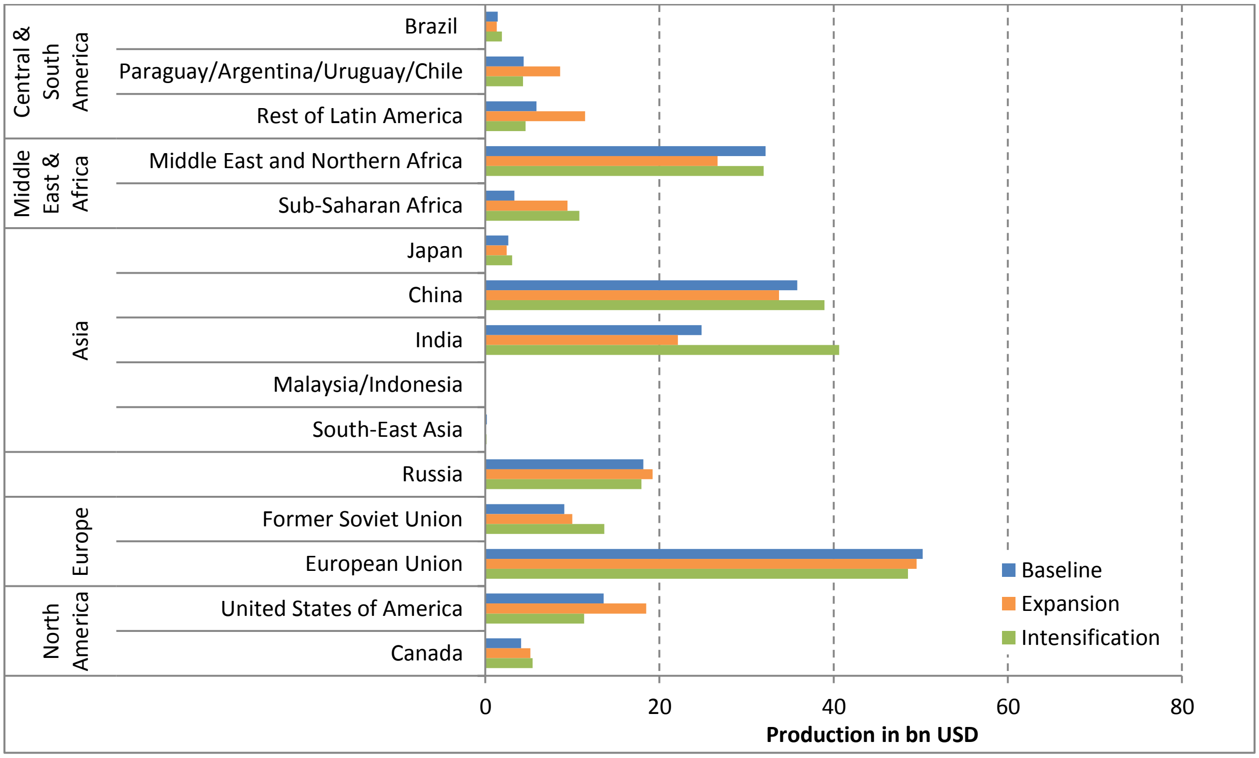

Supplementary Figure 12: Production of wheat in 2030 for the three scenarios.

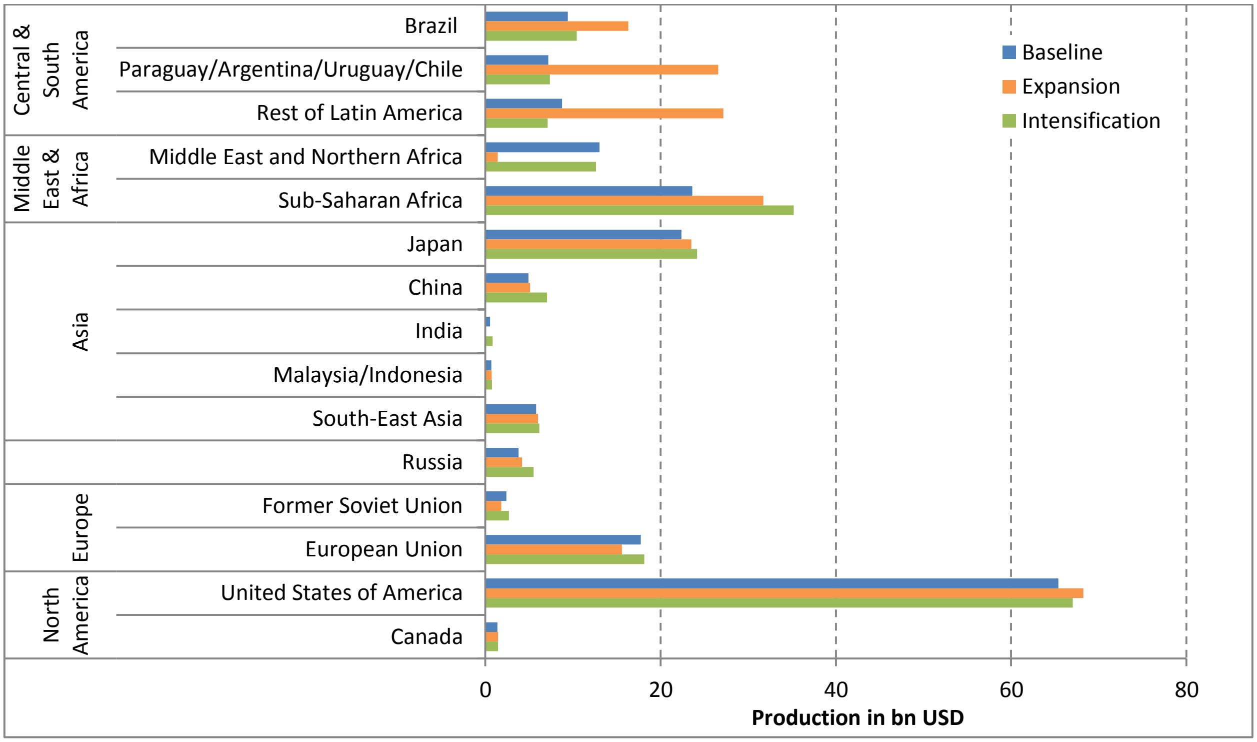

Supplementary Figure 13: Production of maize in 2030 for the three scenarios.

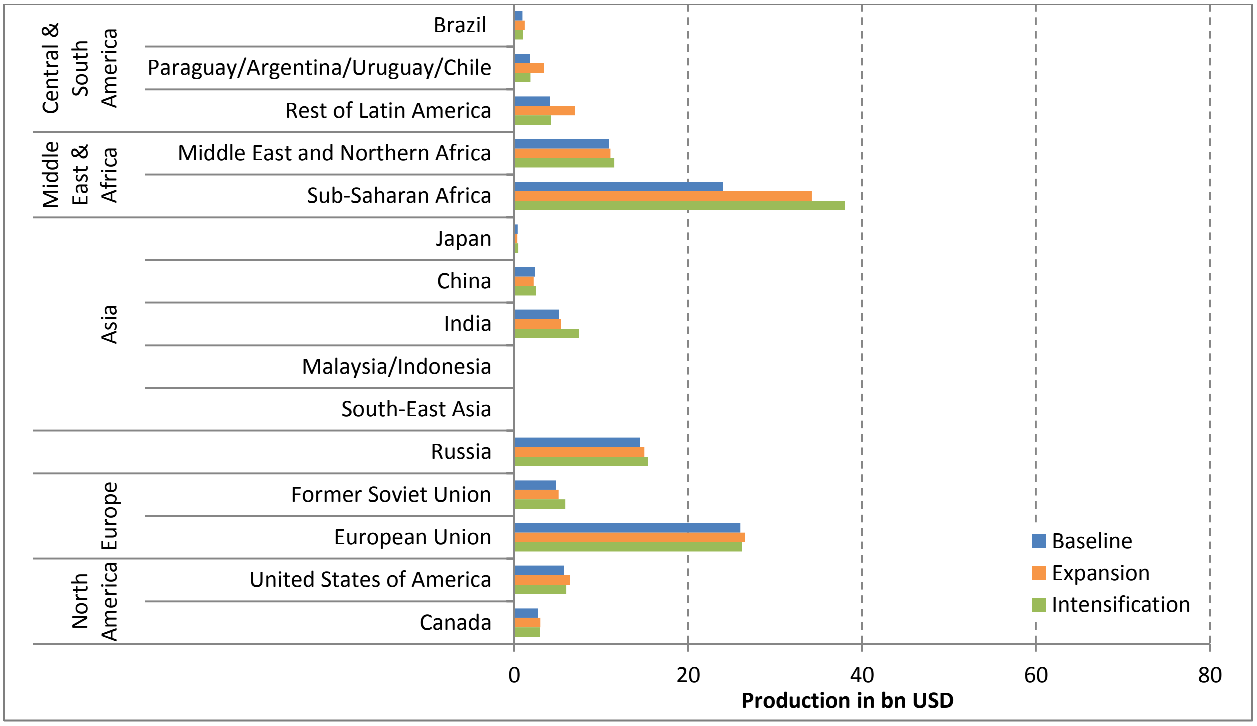

Supplementary Figure 14: Production of rest of cereal grains (total cereal production minus paddy rice, wheat, maize) in 2030 under three scenarios.

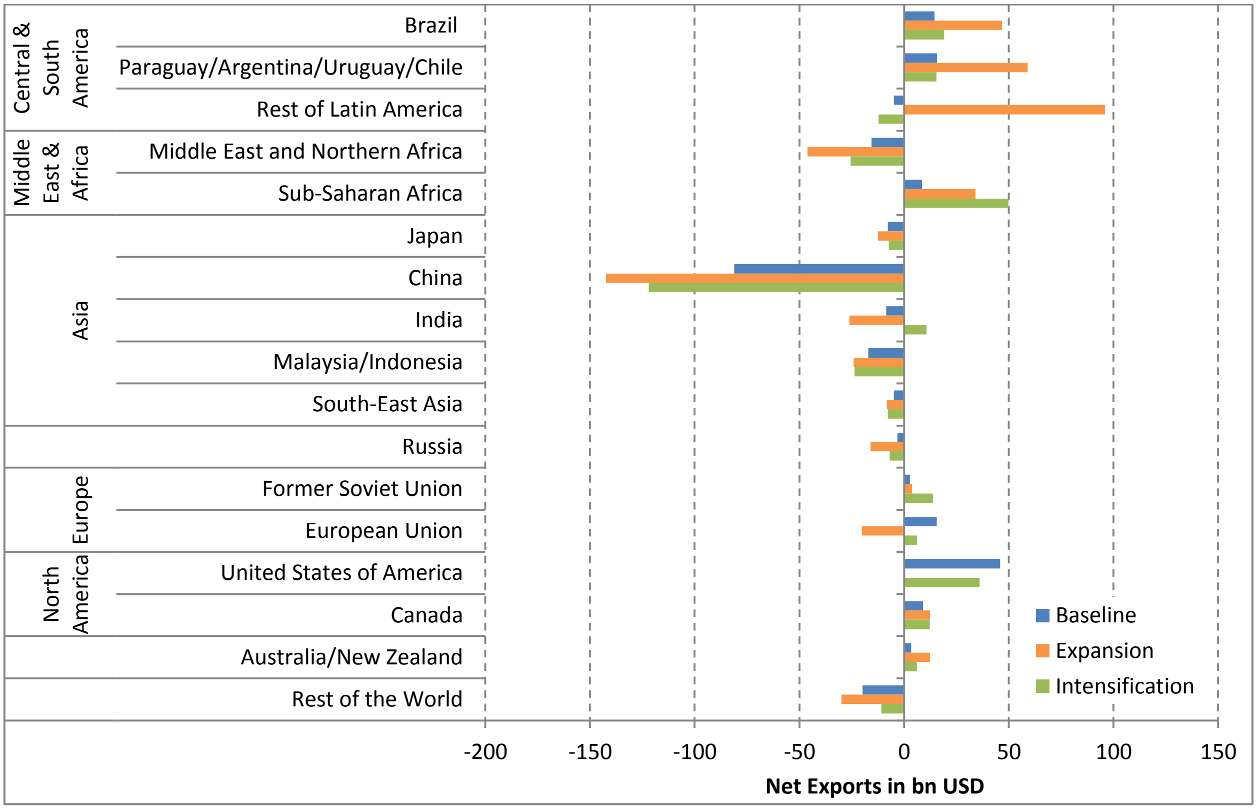

Supplementary Figure 15: Net exports of crops by world region in 2030 under different scenarios. Net exports are exports minus imports; hence negative net exports are equivalent to positive net imports.

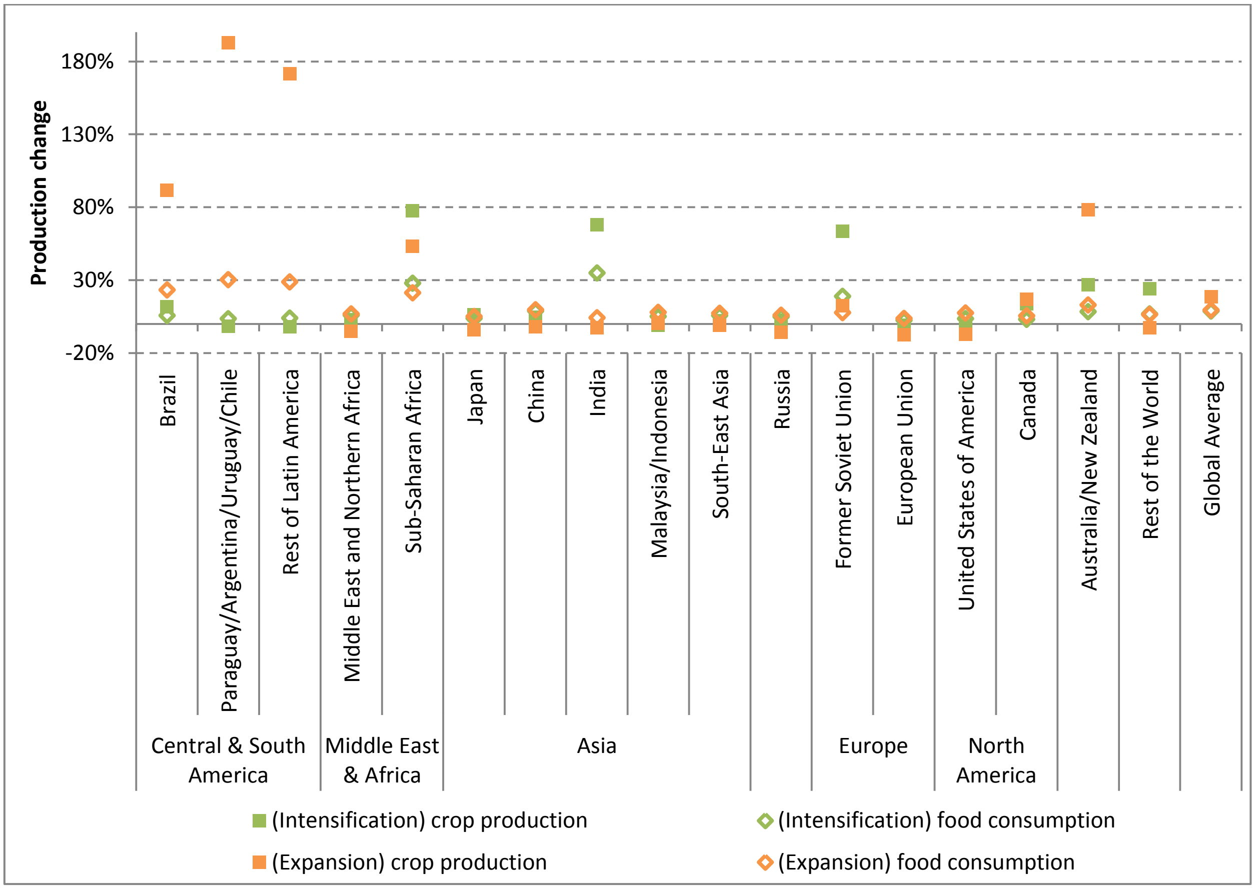

Supplementary Figure 16: Percentage changes in crop production and food consumption by region in 2030 compared to reference scenario.

Supplementary Note 5: Hotspot Analysis

We used information on global patterns of endemism richness (Supplementary Fig. 17) calculated from the International Union for Conservation of Nature (IUCN) and Birdlife databases[40], in order to identify hotspots where biodiversity could be most affected by potential future cropland expansion and intensification. The biodiversity data were aggregated at a 55 km equal area grid, while the integrated expansion and intensification potential data were compiled at 5 arc minutes spatial resolution. Therefore, to conduct the hotspot analyses, we resampled the dataset for integrated expansion and intensification potential to the coarser resolution of the two datasets, summing up the area for expansion and intensification potential in each 55 km grid cell. Two methods were used to identify agriculture-biodiversity hotspots: Local indicator of spatial association and quantile overlay.

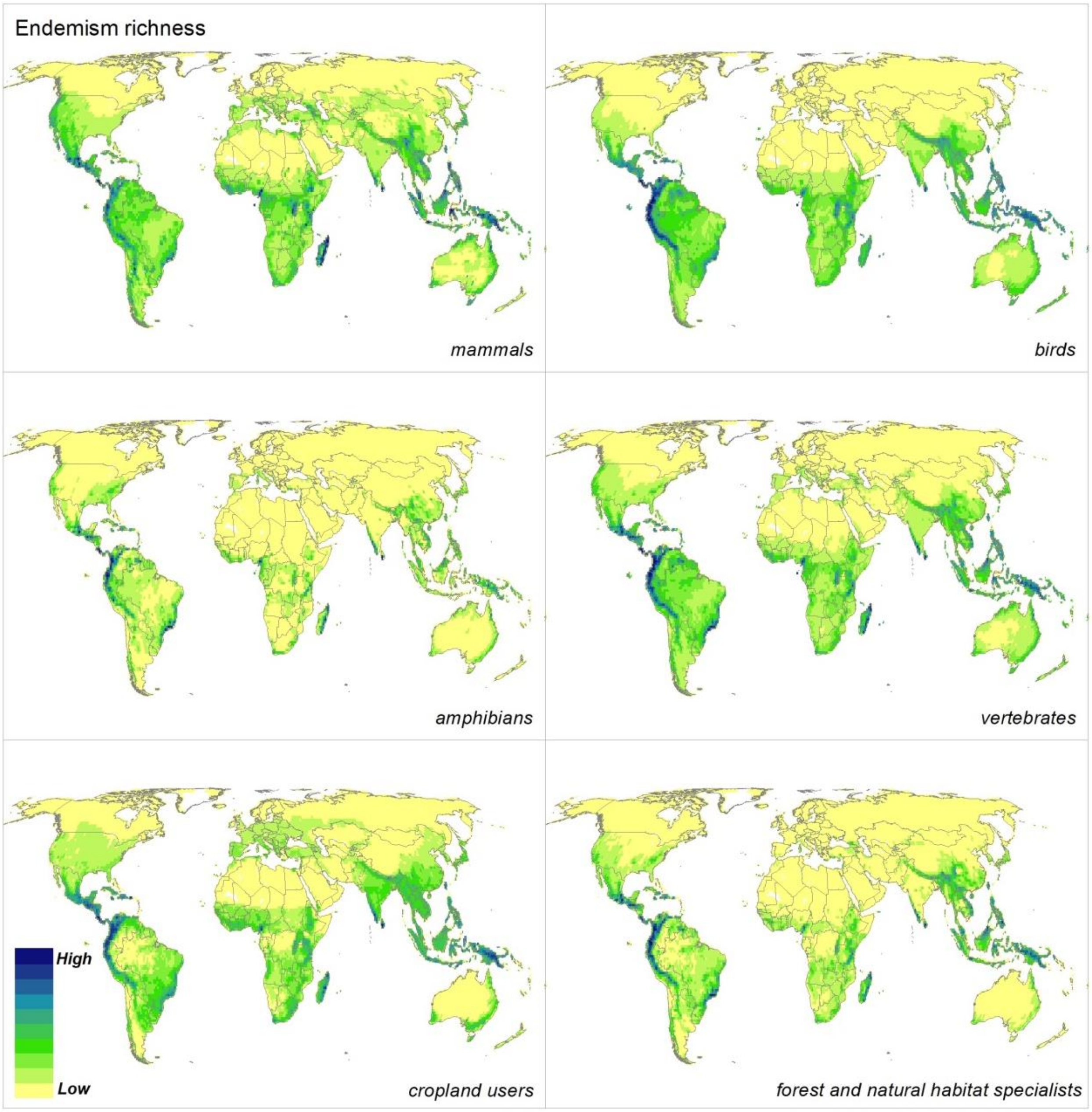

Supplementary Figure 17: Endemism richness data used for hotspot analyses: for mammals, birds, amphibians and all three vertebrate groups together, and for cropland users versus forest and natural habitat specialists.

LISA Analysis

LISA (local indicator of spatial association) represents a local version of the correlation coefficient and shows how the nature and strength of the association between two variables varies across a study area. The method allows for the decomposition of global indicators, such as Moran's I, into the contribution of each individual observation (e.g. a grid cell), while giving an indication of the extent of significant spatial clustering of similar values around that observation.

Using OpenGeoDa version 1.2.0[41], we calculated the local Moran's I statistic of spatial association for each grid cell as:

$$I_i = \frac{x_i - \bar{x}}{s^2} \sum_{j=1, j \neq i}^{n} w_{ij} \left(y_j - \bar{y} \right)$$

where x_i and y_j are values of variable x (i.e. cropland expansion or intensification potentials) and variable y (i.e. endemism richness) for grid cells i and j, respectively, $\bar{x}$ and $\bar{y}$ are the means of the variables, w_{ij} is the spatial weight between cell i and j inversely proportional to Euclidean distance between the two cells, and s^2 is the variance.

Based on the values of local Moran's I, we identified and mapped four types of spatial associations: (1) high-high, (2) low-low, (3) high-low, and (4) low-high clustering. The high-high clusters are spatial hotspots of potential conflict where locations with high values of cropland expansion or intensification potentials are significantly associated with high values of endemism richness. The low-low clusters are spatial cold spots in which locations with low values of cropland expansion or intensification potentials are significantly associated with low values of endemism richness. High-low and low-high clusters represent areas where the spatial association between the variables is negative (inverse). The high-low regions might hold a high potential for production increase either by intensification or expansion but are not in conflict with biodiversity protection. Accordingly, they might be suitable for a sustainable expansion or intensification of cropland. The low-high regions are areas with high endemism richness but rather low estimated potential for agricultural production growth and therefore may be especially suitable for conservation of biodiversity. The strength of the association was measured at the 0.05 significance level using a Monte Carlo randomization procedure based on 499 permutations.

Quantile Overlay Analysis

In a further analysis, we delineated the 'hottest hotspots' of potential future conflict between biodiversity and future agricultural production by extracting the top 5, 10, 15, 20, 25, and 30th percentile of the data distribution for the three variables (integrated expansion potential, integrated intensification potential, and endemism richness)[42]. The different percentiles were used to consider the interplay of the different variables when increasing the percentage shares steadily. Intersecting these top values, we pinpointed the top pressure regions where biodiversity is potentially most threatened by near-future cropland expansion, intensification or both (Supplementary Fig. 18, Supplementary Tables 3, 5). The quantile overlay also allowed for identifying the most suitable regions for sustainable expansion or intensification of cropland with minimal impacts on global biodiversity hotspots. To account for the different effects that agricultural intensification can have on species with different habitat requirements, we examined

quantile overlays also separately for species using cropland as a regular or marginal habitat versus forest and natural habitat specialists (Supplementary Fig. 19).

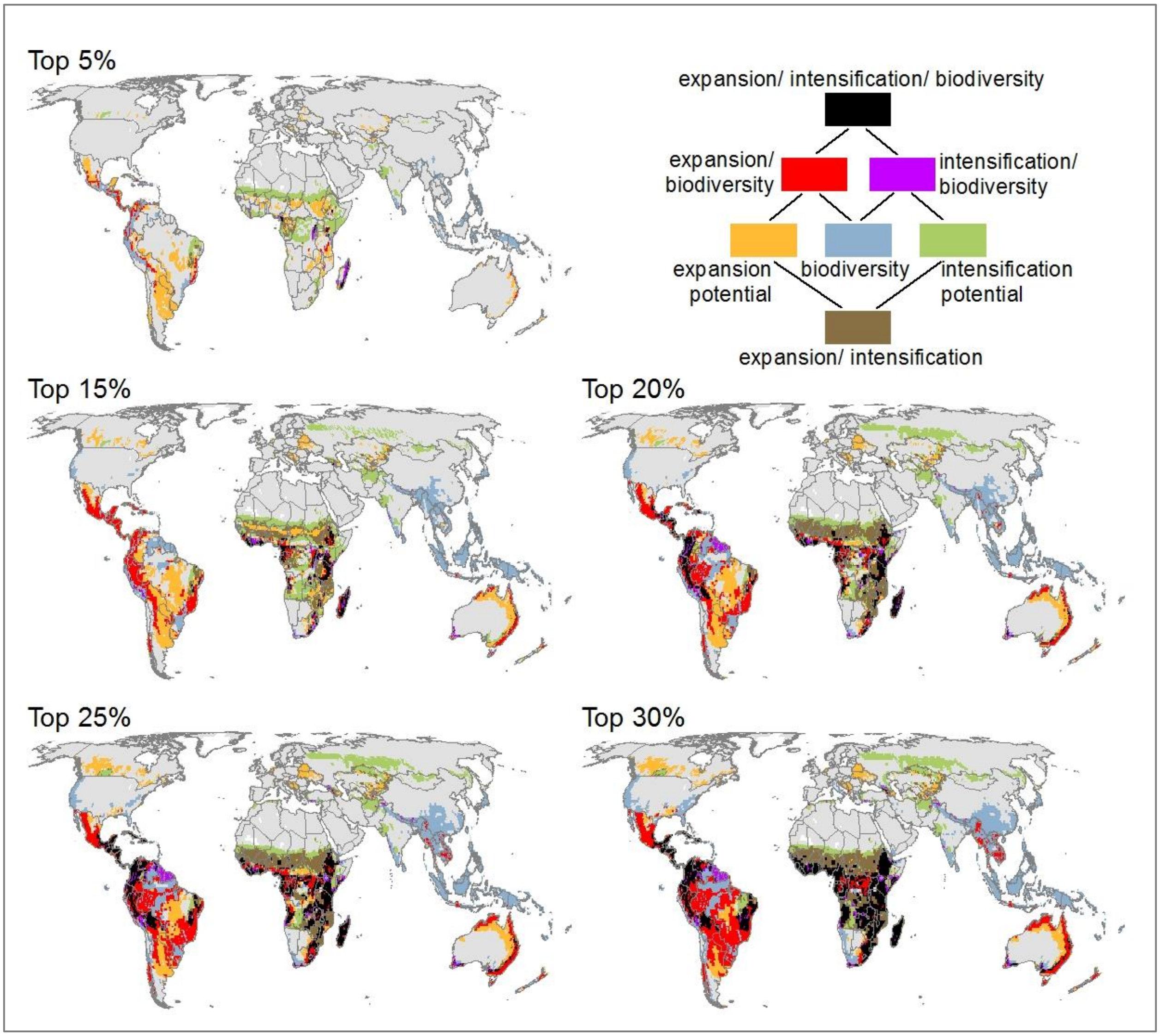

Supplementary Figure 18: Quantile overlay of the top 5, 15, 20, 25 and 30th percentile of expansion potential, intensification potential and endemism richness. (The map for the top 10th percentile is shown in Figure 4 of the main text of the manuscript). The red areas highlight the hottest hotspots, where high biodiversity may be particularly threatened by future cropland expansion. The purple areas highlight the hottest hotspots, where high biodiversity may be particularly affected by future cropland intensification. The black areas pinpoint places where high biodiversity is particularly threatened by both agricultural pathways simultaneously. The orange, green and blue colors indicate regions where the top percentile of expansion potential, intensification potential and biodiversity do not overlap with the same percentile of any other of data, while the brown areas indicate regions where the top percentile of expansion and intensification potential overlap without overlapping the top percentile areas of biodiversity.

Supplementary Table 3: Areas in km² for the different classes of the quantile overlay analysis for the 5th to 30th percentile in 5 percent steps.

Percentile	exp	int	exp&bio	int&bio	exp&int	exp&int&bio
5	3,014,519	784,914	343,678	29,798	311,542	50,749
10	4,305,254	995,747	1,279,052	63,200	1,659,063	422,704
15	5,405,571	894,051	2,913,307	78,275	2,270,574	1,036,588
20	4,666,731	929,382	3,779,670	108,584	4,012,409	3,201,446
25	4,172,568	1,103,166	5,555,240	112,553	4,150,201	5,559,496
30	2,969,232	1,042,432	7,174,946	119,713	4,362,820	8,937,081

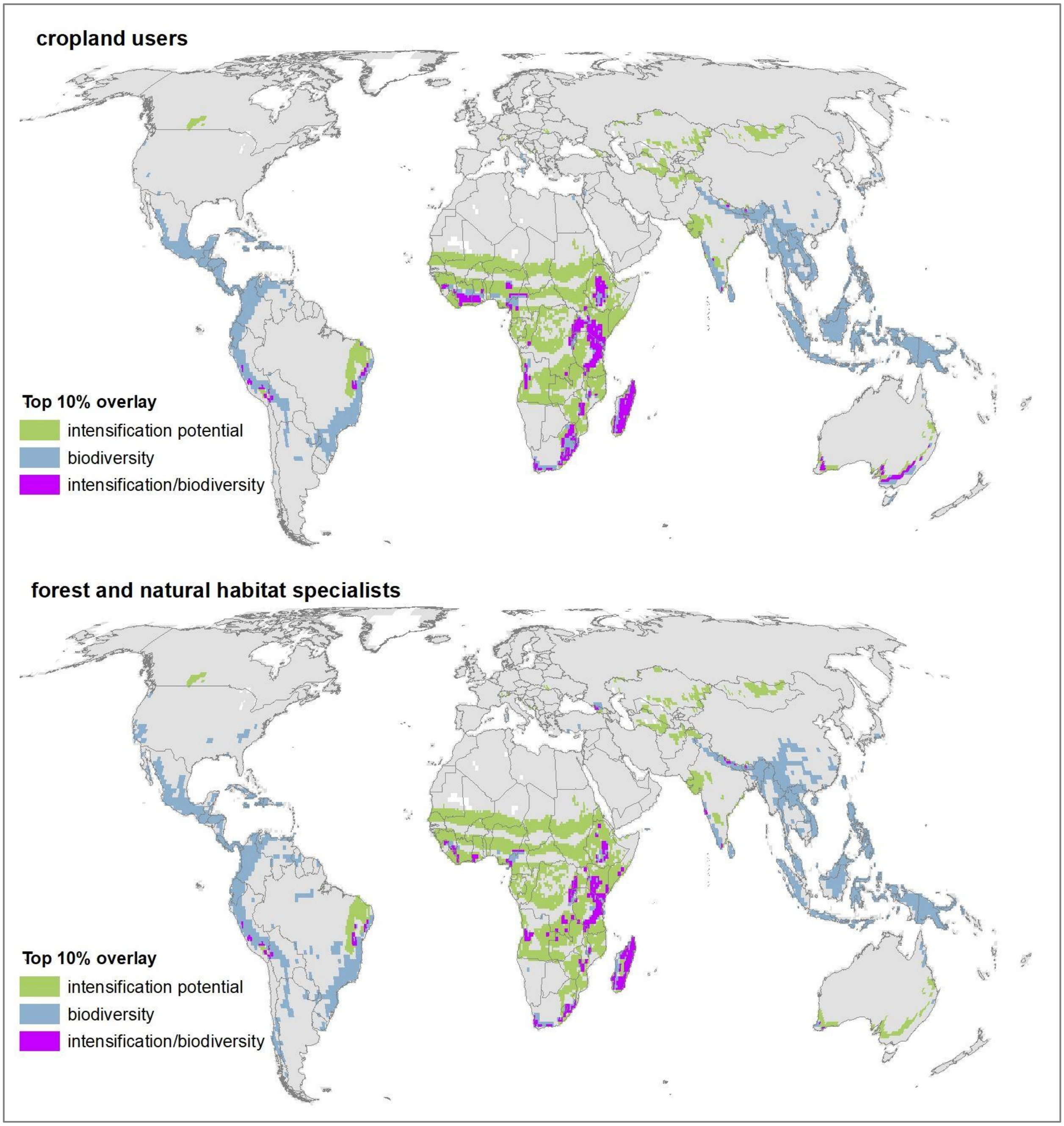

Supplementary Figure 19: Quantile overlay of the top 10th percentile of intensification potential and endemism richness separately for cropland users versus forest and natural habitat specialists. The purple areas highlight the hottest hotspots, where high biodiversity may be particularly affected by future cropland intensification. The green and blue colors indicate regions where the top percentiles of intensification potential and biodiversity do not overlap.

Supplementary Table 4: Areas in km² for the different classes of the quantile overlay analysis for the 5th to 30th percentile in 5 percent steps, differentiated between cropland users and forest specialists & natural habitats.

Percentile	int&bio cropland users	int&bio forest specialists & natural habitats	exp&bio cropland users	exp&bio forest specialists & natural habitats
5	45,012	19,598	415,820	316,930
10	160,713	68,497	1,035,436	1,247,434
15	200,072	95,716	2,388,998	2,673,880
20	302,605	136,459	3,342,219	3,502,777
25	384,016	132,497	4,485,528	4,846,455
30	488,710	182,498	5,145,239	5,521,722

Supplementary Table 5: Areas in km² for the different classes of the quantile overlay analysis for the 10th percentile for different regions. Expansion and intensification areas basically cannot overlap, but they may coexist within a grid cell (55km²). Within the classes exp&int and exp&int&bio a further distinction is made between the coexisting areas of expansion and intensification, based on the available subscale information for expansion and intensification potentials.

Region	exp	int	exp&bio	int&bio	exp&int	exp&int&bio
Global	4,305,254	995,747	1,279,052	63,200	1,659,063<br>1,329,125 (exp)<br>330,176 (int)	422,704<br>352,876 (exp)<br>69,784 (int)
Brazil	871,759	24,823	169,062	368	117,034<br>92,692 (exp)<br>24,345 (int)	17349<br>14,158 (exp)<br>3,192 (int)
Paraguay/Argentina/ Uruguay/Chile	1,176,966	0	136,703	0	0	0
Rest of Latin America	765,845	1,544	767,999	1,824	0	3552<br>3,144 (exp)<br>408 (int)
Middle East and Northern Africa	278	273	0	0	2<br>1 (exp)<br>1 (int)	0
Sub-Saharan Africa	894,178	673,300	129,203	56,906	1,453,146<br>1,169,800 (exp)<br>283,578 (int)	400,220<br>334,380 (exp)<br>65,796 (int)
Japan	423	0	0	0	0	0
China	135	141	0	1	103<br>73 (exp)	0

					30 (int)	
India	0	150,656	0	1,650	0	0
Malaysia/Indonesia	126	0	554	0	0	0
South-East Asia	0	0	3	0	0	0
Russia	1,832	928	0	0	399<br>396 (exp)<br>3 (int)	0
Former Soviet Union	97,916	32,901	0	0	35,620<br>24,973 (exp)<br>10,650 (int)	0
European Union	2,223	23	0	0	178<br>131 (exp)<br>47 (int)	0
United States of America	243	191	0	0	0	0
Canada	102,504	29,962	0	0	0	0
Australia/New Zealand	390,763	66,220	75,528	2,380	52,553<br>41,053 (exp)<br>11,502 (int)	1,582<br>1,194 (exp)<br>388 (int)
Rest of the World	61	14,786	0	71	28<br>6 (exp)<br>12 (int)	0

Supplementary Note 6: Overlap of expansion and intensification potentials with protected areas

According to the world database on protected areas (WDPA)[43], globally an area of 11,470 million km² is reported to be under protection without counting coastal and marine areas[43]. While 39% are strictly protected (IUCN category Ia, Ib, II), 61% are less strictly protected (category III-VI), which means that agriculture is conditionally allowed. There is a total discrepancy in the WDPA dataset of 1.16 million km² between the reported protected areas and the area calculated from the reported polygons (GIS area). In the further course of this analysis, we used the GIS area for further calculations.

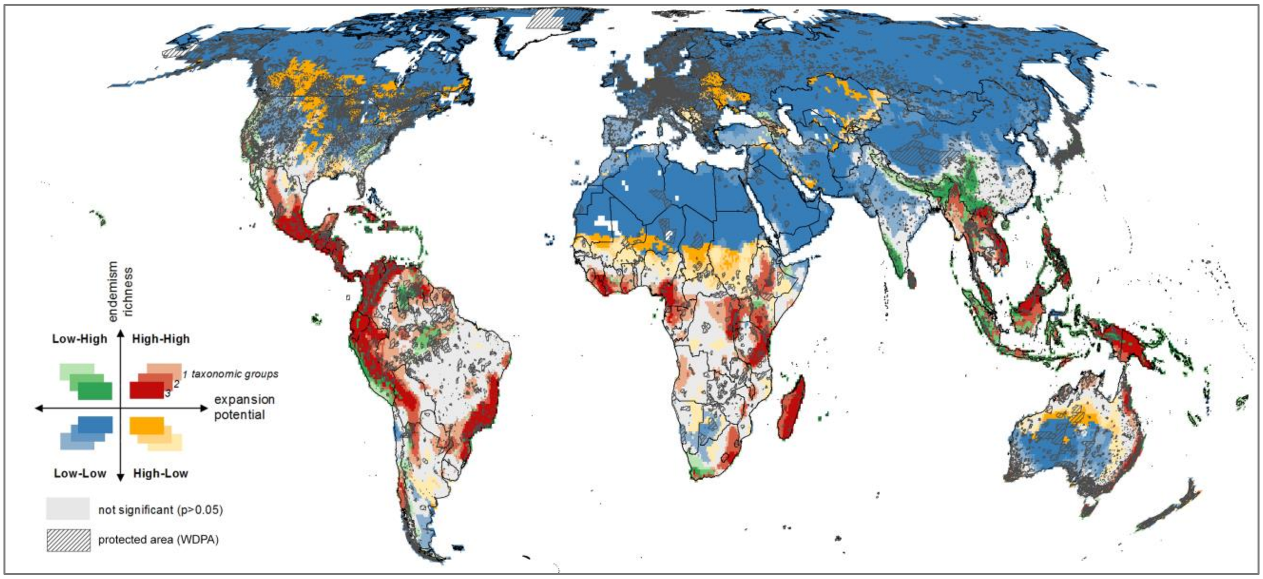

Supplementary Figure 20: Overlap of expansion areas based on the LISA analysis with WDPA protected areas (categories I-VI).

The overlap of the expansion and intensification hotspots from the LISA analysis with the WDPA showed that 26.2% of the intensification hotspots are currently protected, while only 13.2% of the expansion hotspots are protected (Supplementary Fig. 20). In both cases, about 45% of this area is strictly protected and 55% is less strictly protected.

The overlap of the WDPA with the quantile analysis using the 10th percentile threshold for the identification of the hottest hotspots showed that a total area of 625,000 km² threatened by expansion or intensification is designated as protected area. The top 10% areas for expansion correspond to 7.3 mio. km², of which 18% overlay with the top 10% of global biodiversity. Of this area, 65% are not yet under protection, while 19% are already strictly protected, and another 16% are less strictly protected. The top 10% areas for intensification correspond to 1.5 mio. km², of which 4% overlay with the top 10% of global biodiversity. Of this area, 92% are already protected (28% strictly, 64% less strictly), while only 8% remain not protected.

Supplementary Note 7: Policy Scenario: Impact of excluding protected areas from expansion potentials on agricultural markets

In order to analyze a policy where governments enforce that additional crop production is not allowed in protected areas but expansion still takes place at the top 10% area, we restricted expansion only to non-protected areas but still expanded the same global area of 7.3 million km² as in the expansion scenario. Therefore, we excluded the WDPA categories I – VI from the expansion potential and allocated the next best non-protected area until the same global

expansion area was reached. This was done in order to be comparable to the expansion scenario, where no restriction took place. The additional policy scenario is called 'restricted expansion'.

The impacts of restricted expansion are different across regions (Supplementary Fig. 21). Strongest absolute reductions in expansion areas occurred in Sub-Saharan Africa (70,000 km^2), Rest of Latin America, and Australia/New Zealand (13,700 km^2 each), while additional expansion took place in Brazil (76,600 km^2) and Canada (10,500 km^2). The relative changes of expansion area between the two expansion scenarios are relatively small: amongst the regions with expansion areas greater than 100,000 km^2, the changes in the restricted expansion scenario compared to the unrestricted expansion scenario range between +10% and -3%.

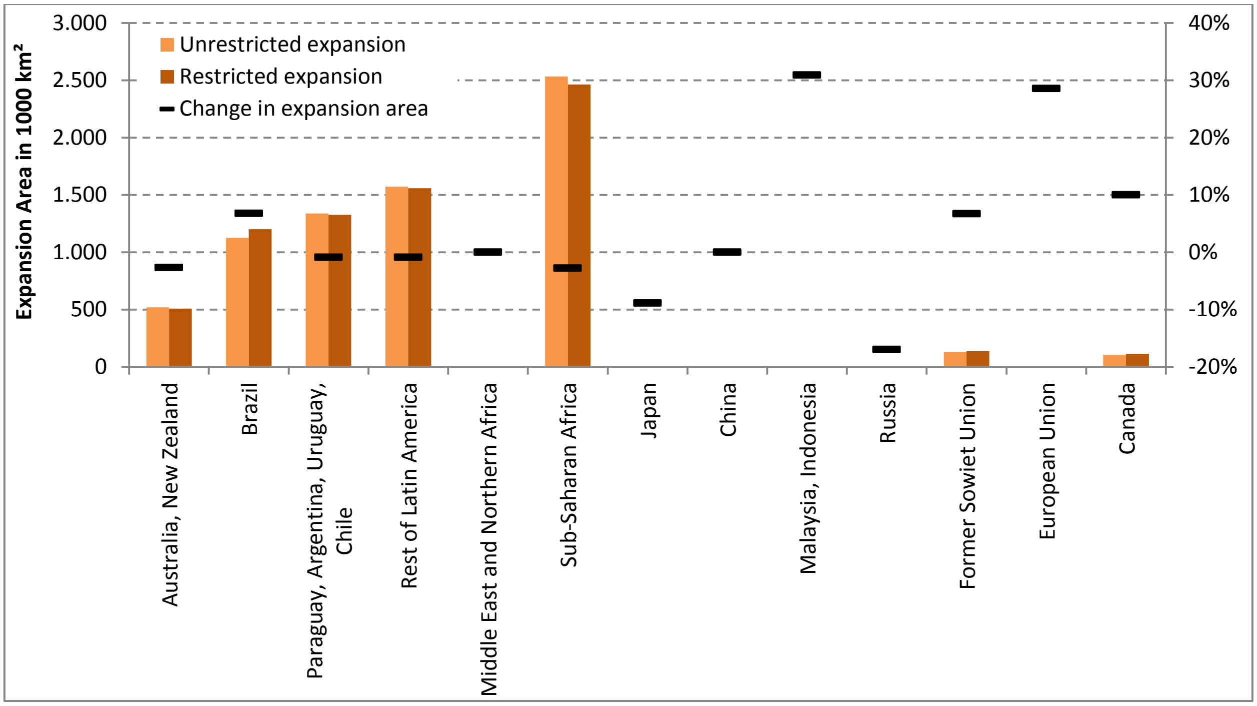

Supplementary Figure 21: Area used for expansion with and without policy restrictions to allow expansion into protected areas. Bars indicate the expansion potentials in 1,000 km², the black dashes illustrate the change between the unrestricted and restricted expansion scenario. Results are only shown for regions with expansion area greater than 100.000 km².

The policy scenario 'restricted expansion' had only minor implications on agricultural markets and trade flows. As illustrated in Supplementary Fig. 22, the restricted expansion scenario is mirrored by changes in crop production: Crop production in Australia/New Zealand decreased by 3 percentage points in the restricted scenario compared to the unrestricted scenario, since less land was utilized for expansion due to the implemented policy assumptions. Contrary, Brazil increased crop production by 4 percentage points, since more land was used for expansion. This

can be explained by Brazil's larger share of the global top 10% expansion area in the restricted expansion scenario. More production resulted in more net exports of crops in Brazil and Canada (Supplementary Fig. 23). Differences in price changes were comparable under the two scenarios. Hence, the policy scenario caused some shifts of crop production amongst regions, but global trade adjusted these differences.

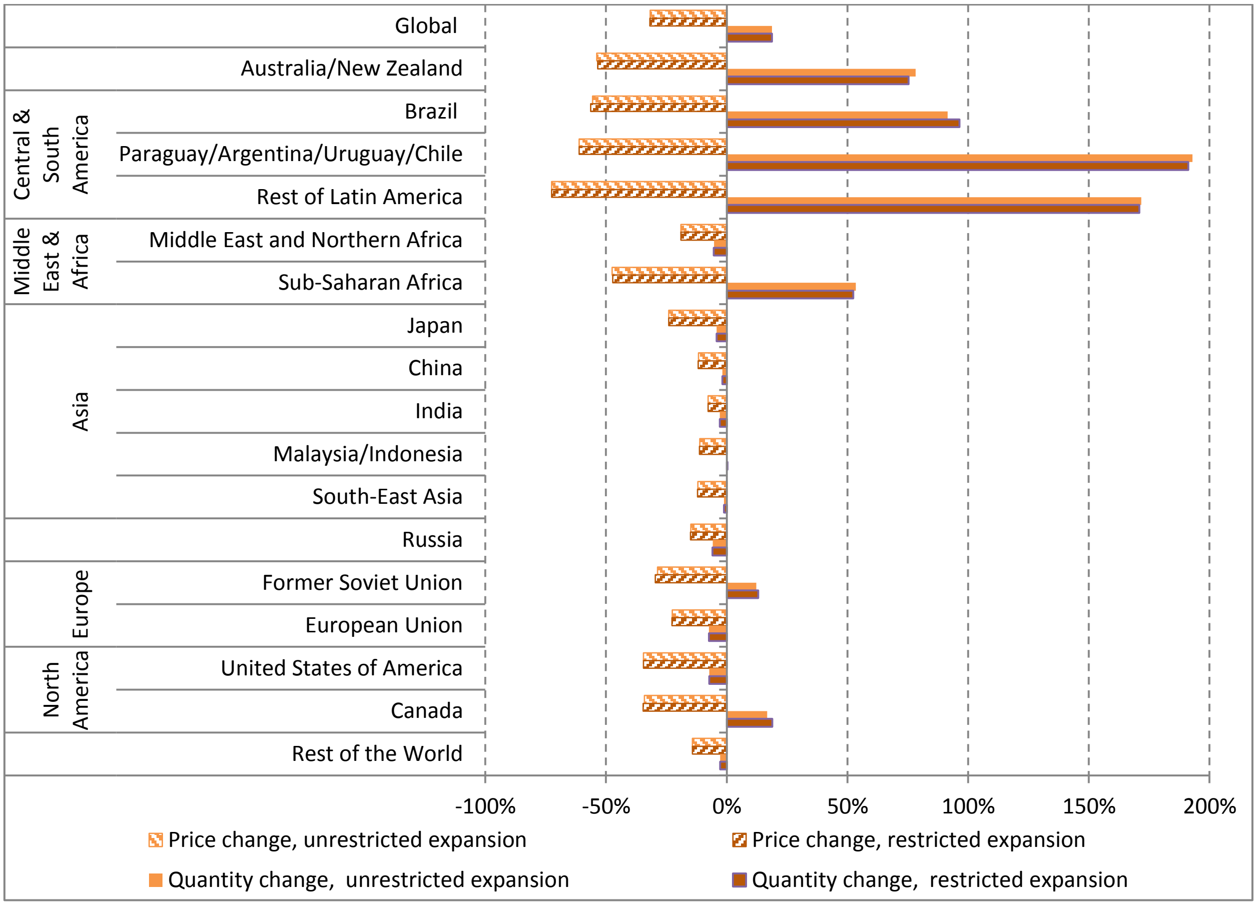

Supplementary Figure 22: Change in crop production and prices under restricted expansion and unrestricted expansion scenarios compared to the reference scenario in 2030.

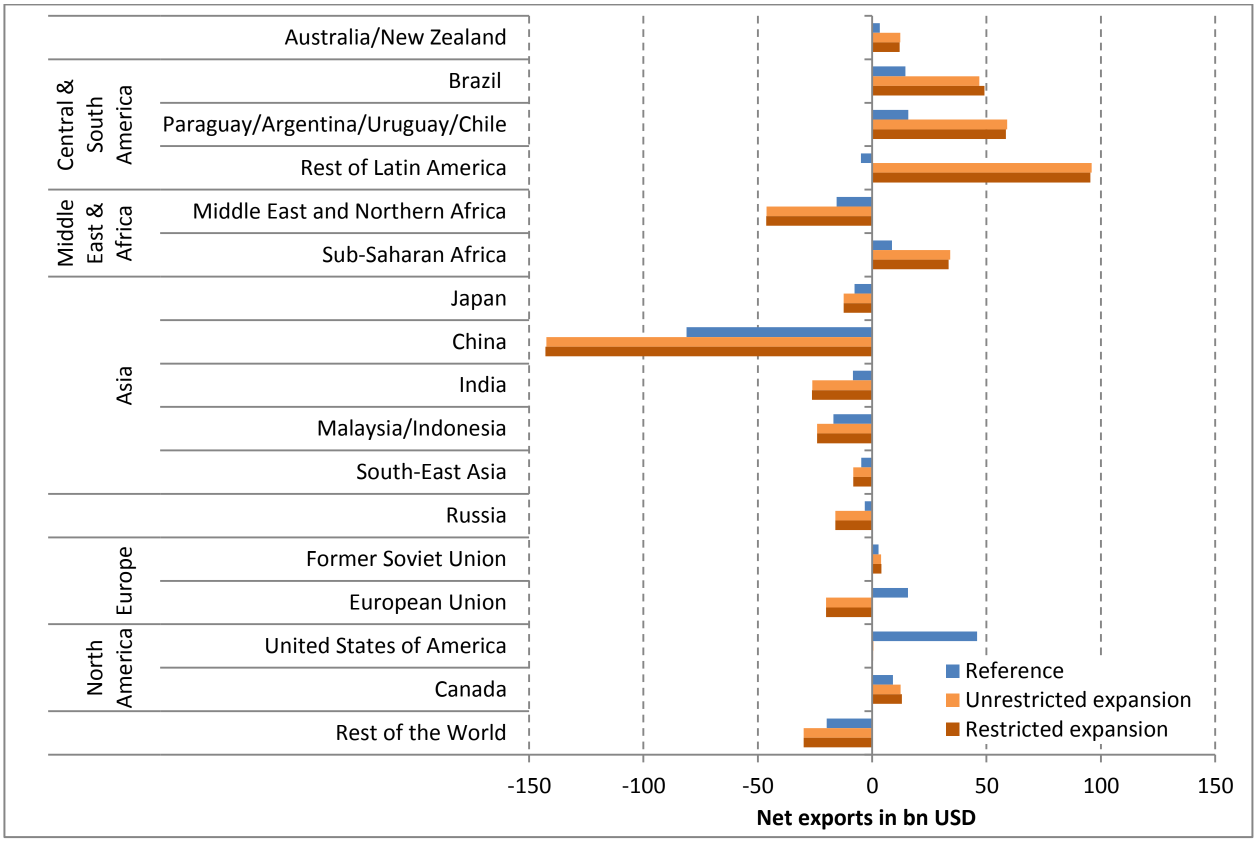

Supplementary Figure 23: Net exports of crops by world region in 2030 under different scenarios.

Supplementary Note 8: Data sources

- GTAP data (licence required; Narayanan, B., Aguiar, A. & McDougall, R. Global Trade, Assistance, and Production: The GTAP 8 Data Base. (ed Center for Global Trade Analysis) (Purdue University, 2012).
- UN population data: United Nations, Department of Economic and Social Affairs, Population Division. World Population Prospects: The 2012 Revision, Highlights and Advance Tables. Working Paper No. ESA/P/WP.228, (2013).
- GDP data from OECD: OECD Environmental Outlook to 2050: The Consequences of Inaction. (OECD Publishing, Paris, 2012).
- Global biodiversity data: global range maps for 19,978 species of birds, mammals and amphibians:
    - BirdLife Data Zone. http://www.birdlife.org/datazone/home (2012).
    - IUCN. The IUCN Red List of Threatened Species. http://www.iucnredlist.org/technical-documents/spatial-data (2012).

    - Egli, L., Meyer, C., Scherber, C., Kreft, H. & Tscharntke, T. Winners and losers of national and global efforts to reconcile agricultural intensification and biodiversity conservation. Global Change Biol 24, 2212-2228, doi:10.1111/gcb.14076 (2018).
- Land suitability data is based on the approach by Zabel, F., Putzenlechner, B. & Mauser, W. Global Agricultural Land Resources – A High Resolution Suitability Evaluation and Its Perspectives until 2100 under Climate Change Conditions. PLoS ONE 9, e107522, doi:10.1371/journal.pone.0107522 (2014).
- Land use data:
    - Monfreda, C., Ramankutty, N. & Foley, J. A. Farming the planet: 2. Geographic distribution of crop areas, yields, physiological types, and net primary production in the year 2000. Global Biogeochemical Cycles 22, GB1022, doi:10.1029/2007GB002947 (2008).
    - Ramankutty, N., Evan, A. T., Monfreda, C. & Foley, J. A. Farming the planet: 1. Geographic distribution of global agricultural lands in the year 2000. Global Biogeochemical Cycles 22, GB1003, doi:10.1029/2007GB002952 (2008).
    - Land use/cover data: ESA. Land Cover CCI Version 2. http://maps.elie.ucl.ac.be/CCI/viewer/index.php (2014).
    - Global irrigated areas: Meier, J., Zabel, F. & Mauser, W. A global approach to estimate irrigated areas – a comparison between different data and statistics. Hydrol. Earth Syst. Sci 22, 1119-1133, doi:10.5194/hess-22-1119-2018 (2018).
    - Siebert, S., Henrich, V., Frenken, K. & Burke, J. Global Map of Irrigation Areas version 5. (Rheinische Friedrich-Wilhelms-University, Bonn, Germany / Food and Agriculture Organization of the United Nations, Rome, Italy, 2013).
- Data on protected areas: UNEP-WCMC. World Database on Protected Areas User Manual 1.0. (UNEP-WCMC, Cambridge, UK, 2015).
- Soil data: FAO, IIASA, ISRIC, ISSCAS & JRC. Harmonized World Soil Database (version 1.21). (FAO, Rome, Italy and IIASA, Laxenburg, Austria, 2012).
- Topography data: Farr, T. G. et al. The Shuttle Radar Topography Mission. Reviews of Geophysics 45, RG2004, doi:10.1029/2005RG000183 (2007).

Supplementary References

1 Hank, T. B., Bach, H. & Mauser, W. Using a Remote Sensing-Supported Hydro-Agroecological Model for Field-Scale Simulation of Heterogeneous Crop Growth and Yield: Application for Wheat in Central Europe. *Remote Sensing* **7**, 3934-3965, doi:10.3390/rs70403934 (2015).

2 Mauser, W. *et al.* Global biomass production potentials exceed expected future demand without the need for cropland expansion. *Nat Commun* **6**, doi:10.1038/ncomms9946 (2015).

3 Mauser, W. & Bach, H. PROMET - Large scale distributed hydrological modelling to study the impact of climate change on the water flows of mountain watersheds. *Journal of Hydrology* **376**, 362-377, doi:10.1016/j.jhydrol.2009.07.046 (2009).

4 Mauser, W. *et al.* PROMET - Processes of Mass and Energy Transfer - An Integrated Land Surface Processes and Human Impacts Simulator for the Quantitative Exploration of Human-Environment Relations. Part 1: Algorithms Theoretical Baseline Document. http://www.geographie.uni-muenchen.de/department/fiona/forschung/projekte/promet_handbook/index.html. 167 (Department of Geography, Munich, 2015).

5 Farquhar, G. D., Caemmerer, S. & Berry, J. A. A biochemical model of photosynthetic CO2 assimilation in leaves of C3 species. *Planta* **149**, 78-90, doi:10.1007/BF00386231 (1980).

6 Ball, J. T., Woodrow, I. & Berry, J. in *Progress in Photosynthesis Research* (ed J. Biggins) Ch. 48, 221-224 (Springer Netherlands, 1987).

7 Yin, X. & van Laar, H. *Crop Systems Dynamics. An Ecophysiological Simulation Model for Genotype-By-Environment Interactions*. (Wageningen Academic Publishers, 2005).

8 Klepper, G. & Peterson, S. Marginal abatement cost curves in general equilibrium: The influence of world energy prices. *Resour Energy Econ* **28**, 1-23, doi:10.1016/j.reseneeco.2005.04.001 (2006).

9 Springer, K. Climate Policy in a Globalizing World - A CGE Model with Capital Mobility and Trade. *JEcon* **82**, 89-93, doi:10.1007/s00712-003-0056-4 (2004).

10 Stone, R. Linear Expenditure Systems and Demand Analysis: An Application to the Pattern of British Demand. *The Economic Journal* **64**, 511-527, doi:10.2307/2227743 (1954).

11 Narayanan, B., Aguiar, A. & McDougall, R. Global Trade, Assistance, and Production: The GTAP 8 Data Base. (ed Center for Global Trade Analysis) (Purdue University, 2012).

12 Baldos, U. L. C. & Hertel, T. W. Development of a GTAP 8 Land Use and Land Cover Data Base for Years 2004 and 2007. In: *GTAP Research Memorandum No. 23* (2012).

13 Calzadilla, A., Delzeit, R. & Klepper, G. Assessing the Effects of Biofuel Quotas on Agricultural Markets. In: *The WSPC Reference on Natural Resources and Environmental Policy in the Era of Global Change* (ed Calzadilla et al.) (World Scientific, 2016).

14 Calzadilla, A., Delzeit, R. & Klepper, G. DART-BIO: Modelling the interplay of food, feed and fuels in a global CGE model. (Kiel Institute for the World Economy, Kiel, Germany, 2014).

15 Hilderink, H. B. M. PHOENIX plus: the population user support system version 1.0 (2000).

16 OECD. *OECD Environmental Outlook to 2050: The Consequences of Inaction*. (OECD Publishing, Paris, 2012).

17 United Nations, Department of Economic and Social Affairs, Population Division. *World Population Prospects: The 2012 Revision, Highlights and Advance Tables*. Working Paper No. ESA/P/WP.228, (2013).

18 van Dijk, M., Philippidis, G. & Woltjer, G. Catching up with history: A methodology to validate global CGE models. (LEI Wageningen UR, 2016).

19 Banse, M., van Meijl, H., Tabeau, A. & Woltjer, G. Will EU biofuel policies affect global agricultural markets? *European Review of Agricultural Economics* **35**, 117-141, doi:10.1093/erae/jbn023 (2008).

20 Bouët, A., Dimaranan, B. V. & Valin, H. Modeling the global trade and environmental impacts of biofuel policies. (IFPRI, Washington D.C., 2010).

21 Laborde, D. & Valin, H. Modeling land-use changes in a global CGE: Assessing the EU biofuel mandates with the MIRAGE-BioF model. *Climate Change Economics* **03**, 39, doi:10.1142/s2010007812500170 (2012).

22 Robinson, S. *et al.* Comparing supply-side specifications in models of global agriculture and the food system. *Agricultural Economics* **45**, 21-35, doi:10.1111/agec.12087 (2014).

23 Delzeit, R., Klepper, G., Zabel, F. & Mauser, W. Global economic–biophysical assessment of midterm scenarios for agricultural markets—biofuel policies, dietary patterns, cropland expansion, and productivity growth. *Environmental Research Letters* **13**, 025003 (2018).

24 OECD/FAO. OECD-FAO Agricultural Outlook 2016-2025. (OECD Publishing, Paris, 2016).

25 Beurskens, L.W.M., Hekkenberg, M. & Vethman, P. Renewable Energy Projections (ECN-E—10-069). National Renewable Energy Action Plans of the European Member States. European Environmental Agency (2011).

26 Delzeit, R., Zabel, F., Meyer, C. & Václavík, T. Addressing future trade-offs between biodiversity and cropland expansion to improve food security. *Regional Environmental Change* **17**, 1429-1441, doi:10.1007/s10113-016-0927-1 (2017).

27 Zabel, F., Putzenlechner, B. & Mauser, W. Global Agricultural Land Resources – A High Resolution Suitability Evaluation and Its Perspectives until 2100 under Climate Change Conditions. *PLoS ONE* **9**, e107522, doi:10.1371/journal.pone.0107522 (2014).

28 Ramankutty, N., Evan, A. T., Monfreda, C. & Foley, J. A. Farming the planet: 1. Geographic distribution of global agricultural lands in the year 2000. *Global Biogeochemical Cycles* **22**, GB1003, doi:10.1029/2007GB002952 (2008).

29 ESA. Land Cover CCI Version 2. http://maps.elie.ucl.ac.be/CCI/viewer/index.php (2014).

30 Bengtsson, L., Hodges, K. I. & Keenlyside, N. Will Extratropical Storms Intensify in a Warmer Climate? *Journal of Climate* **22**, 2276-2301, doi:10.1175/2008JCLI2678.1 (2009).

31 Bengtsson, L. *et al.* How may tropical cyclones change in a warmer climate? *Tellus A* **59**, 539-561, doi:10.1111/j.1600-0870.2007.00251.x (2007).

32 Jungclaus, J. H. *et al.* Ocean Circulation and Tropical Variability in the Coupled Model ECHAM5/MPI-OM. *Journal of Climate* **19**, 3952-3972, doi:10.1175/JCLI3827.1 (2006).

33 Hijmans, R. J., Cameron, S. E., Parra, J. L., Jones, P. G. & Jarvis, A. Very high resolution interpolated climate surfaces for global land areas. *International Journal of Climatology* **25**, 1965-1978, doi:10.1002/joc.1276 (2005).

34 FAO, IIASA, ISRIC, ISSCAS & JRC. Harmonized World Soil Database (version 1.21). (FAO, Rome, Italy and IIASA, Laxenburg, Austria, 2012).

35 Farr, T. G. *et al.* The Shuttle Radar Topography Mission. *Reviews of Geophysics* **45**, RG2004, doi:10.1029/2005RG000183 (2007).

36 Meier, J., Zabel, F. & Mauser, W. A global approach to estimate irrigated areas – a comparison between different data and statistics. *Hydrol. Earth Syst. Sci* **22**, 1119-1133, doi:10.5194/hess-22-1119-2018 (2018).

37 Alexandratos, N. & Bruinsma, J. World agriculture towards 2030/2050: the 2012 revision. (FAO, Rome, 2012).

38 Siebert, S., Henrich, V., Frenken, K. & Burke, J. Global Map of Irrigation Areas version 5. (Rheinische Friedrich-Wilhelms-University, Bonn, Germany / Food and Agriculture Organization of the United Nations, Rome, Italy, 2013).

39 Monfreda, C., Ramankutty, N. & Foley, J. A. Farming the planet: 2. Geographic distribution of crop areas, yields, physiological types, and net primary production in the year 2000. *Global Biogeochemical Cycles* **22**, GB1022, doi:10.1029/2007GB002947 (2008).

40 Kier, G. *et al.* A global assessment of endemism and species richness across island and mainland regions. *Proceedings of the National Academy of Sciences* **106**, 9322-9327, doi:10.1073/pnas.0810306106 (2009).

41 Anselin, L., Syabri, I. & Kho, Y. GeoDa: An introduction to spatial data analysis. *Geogr Anal* **38**, 5-22, doi:10.1111/j.0016-7363.2005.00671.x (2006).

42 Ceballos, G. & Ehrlich, P. R. Global mammal distributions, biodiversity hotspots, and conservation. *Proceedings of the National Academy of Sciences* **103**, 19374-19379, doi:10.1073/pnas.0609334103 (2006).

43 UNEP-WCMC. World Database on Protected Areas User Manual 1.0. (UNEP-WCMC, Cambridge, UK, 2015).
