# Supplementary material for: Global impacts of future cropland expansion and intensification on agricultural markets and biodiversity
Source: Nat Commun. 2019 Jun 28;10:2844. doi: 10.1038/s41467-019-10775-z (PMC6598988; doi:10.1038/s41467-019-10775-z)
Supplement: Supplementary file 3 — Reporting Summary [file 41467_2019_10775_MOESM3_ESM.pdf]

nature research

Corresponding author(s): DBPR

Reporting Summary

Nature Research wishes to improve the reproducibility of the work that we publish. This form provides structure for consistency and transparency in reporting. For further information on Nature Research policies, see Authors & Referees and the Editorial Policy Checklist.

Statistical parameters

When statistical analyses are reported, confirm that the following items are present in the relevant location (e.g. figure legend, table legend, main text, or Methods section).

n/a	Confirmed	
☒	☐	The exact sample size (*n*) for each experimental group/condition, given as a discrete number and unit of measurement
☒	☐	An indication of whether measurements were taken from distinct samples or whether the same sample was measured repeatedly
☒	☐	The statistical test(s) used AND whether they are one- or two-sided<br>*Only common tests should be described solely by name; describe more complex techniques in the Methods section.*
☒	☐	A description of all covariates tested
☒	☐	A description of any assumptions or corrections, such as tests of normality and adjustment for multiple comparisons
☒	☐	A full description of the statistics including central tendency (e.g. means) or other basic estimates (e.g. regression coefficient) AND variation (e.g. standard deviation) or associated estimates of uncertainty (e.g. confidence intervals)
☒	☐	For null hypothesis testing, the test statistic (e.g. *F*, *t*, *r*) with confidence intervals, effect sizes, degrees of freedom and *P* value noted<br>*Give P values as exact values whenever suitable.*
☒	☐	For Bayesian analysis, information on the choice of priors and Markov chain Monte Carlo settings
☒	☐	For hierarchical and complex designs, identification of the appropriate level for tests and full reporting of outcomes
☒	☐	Estimates of effect sizes (e.g. Cohen's *d*, Pearson's *r*), indicating how they were calculated
☒	☐	Clearly defined error bars<br>*State explicitly what error bars represent (e.g. SD, SE, CI)*

Our web collection on statistics for biologists may be useful.

Software and code

Policy information about availability of computer code

Data collection	There was no code used for data collection.
Data analysis	GAMS software, code of DART model not pubished but can be provided upon demand.<br>OpenGeoDa software for LISA analysis.<br>ArcGIS software for all spatial overlay analyses.<br>The code for coupling DART and PROMET is available upon request.<br>PROMET is described in published literature cited in the paper. PROMET code is not publically available because of both scientific and commercial use. It may be opened to editor and reviewers under specific NDA agreements.

For manuscripts utilizing custom algorithms or software that are central to the research but not yet described in published literature, software must be made available to editors/reviewers upon request. We strongly encourage code deposition in a community repository (e.g. GitHub). See the Nature Research guidelines for submitting code & software for further information.

Data

Policy information about availability of data

All manuscripts must include a data availability statement. This statement should provide the following information, where applicable:

- Accession codes, unique identifiers, or web links for publicly available datasets
- A list of figures that have associated raw data
- A description of any restrictions on data availability

GTAP data (licence required; Narayanan, B., Aguiar, A. & McDougall, R. Global Trade, Assistance, and Production: The GTAP 8 Data Base. (ed Center for Global Trade Analysis) (Purdue University, 2012).
UN population data: United Nations, Department of Economic and Social Affairs, Population Division. World Population Prospects: The 2012 Revision, Highlights and Advance Tables. Working Paper No. ESA/P/WP.228, (2013).
GDP data from OECD: OECD Environmental Outlook to 2050: The Consequences of Inaction. (OECD Publishing, Paris, 2012).
Global biodiversity data: global range maps for 19,978 species of birds, mammals and amphibians:
BirdLife Data Zone. http://www.birdlife.org/datazone/home (2012).
IUCN. The IUCN Red List of Threatened Species. http://www.iucnredlist.org/technical-documents/spatial-data (2012).
Egli, L., Meyer, C., Scherber, C., Kreft, H. & Tscharntke, T. Winners and losers of national and global efforts to reconcile agricultural intensification and biodiversity conservation. Global Change Biol 24, 2212-2228, doi:10.1111/gcb.14076 (2018).
Land suitability data is based on the approach by Zabel, F., Putzenlechner, B. & Mauser, W. Global Agricultural Land Resources – A High Resolution Suitability Evaluation and Its Perspectives until 2100 under Climate Change Conditions. PLoS ONE 9, e107522, doi:10.1371/journal.pone.0107522 (2014).
Land use data:
Monfreda, C., Ramankutty, N. & Foley, J. A. Farming the planet: 2. Geographic distribution of crop areas, yields, physiological types, and net primary production in the year 2000. Global Biogeochemical Cycles 22, GB1022, doi:10.1029/2007GB002947 (2008).
Ramankutty, N., Evan, A. T., Monfreda, C. & Foley, J. A. Farming the planet: 1. Geographic distribution of global agricultural lands in the year 2000. Global Biogeochemical Cycles 22, GB1003, doi:10.1029/2007GB002952 (2008).
Land use/cover data: ESA. Land Cover CCI Version 2. http://maps.elie.ucl.ac.be/CCI/viewer/index.php (2014).
Global irrigated areas: Meier, J., Zabel, F. & Mauser, W. A global approach to estimate irrigated areas – a comparison between different data and statistics. Hydrol. Earth Syst. Sci 22, 1119-1133, doi:10.5194/hess-22-1119-2018 (2018).
Siebert, S., Henrich, V., Frenken, K. & Burke, J. Global Map of Irrigation Areas version 5. (Rheinische Friedrich-Wilhelms-University, Bonn, Germany / Food and Agriculture Organization of the United Nations, Rome, Italy, 2013).
Data on protected areas: UNEP-WCMC. World Database on Protected Areas User Manual 1.0. (UNEP-WCMC, Cambridge, UK, 2015).
Soil data: FAO, IIASA, ISRIC, ISSCAS & JRC. Harmonized World Soil Database (version 1.21). (FAO, Rome, Italy and IIASA, Laxenburg, Austria, 2012).
Topography data: Farr, T. G. et al. The Shuttle Radar Topography Mission. Reviews of Geophysics 45, RG2004, doi:10.1029/2005RG000183 (2007).

Field-specific reporting

Please select the best fit for your research. If you are not sure, read the appropriate sections before making your selection.

☐ Life sciences ☐ Behavioural & social sciences ☒ Ecological, evolutionary & environmental sciences

For a reference copy of the document with all sections, see nature.com/authors/policies/ReportingSummary-flat.pdf

Ecological, evolutionary & environmental sciences study design

All studies must disclose on these points even when the disclosure is negative.

Study description	We iteratively link a global computable general equilibrium model and a dynamic crop growth model to determine the impacts of land-use intensification and cropland expansion on agricultural markets. Land-use intensification and cropland expansion potentials for 2030 are used to identify spatial associations with areas of high biodiversity value.
Research sample	this does not apply for our scenario analysis.
Sampling strategy	this does not apply for our scenario analysis.
Data collection	we did not collect data ourselves.
Timing and spatial scale	we did not collect data ourselves.
Data exclusions	this does not apply for our scenario analysis.
Reproducibility	this does not apply for our scenario analysis since we do not apply experiments. The scenario runs can be reproduced.
Randomization	this does not apply for our scenario analysis.
Blinding	we did not collect data ourselves.

Did the study involve field work? ☐ Yes ☒ No

Reporting for specific materials, systems and methods

Materials & experimental systems

n/a	Involved in the study
☒	☐ Unique biological materials
☒	☐ Antibodies
☒	☐ Eukaryotic cell lines
☒	☐ Palaeontology
☒	☐ Animals and other organisms
☒	☐ Human research participants

Methods

n/a	Involved in the study
☒	☐ ChIP-seq
☒	☐ Flow cytometry
☒	☐ MRI-based neuroimaging
